# Beyond Cartilage‐Inspired Supramolecular Polyurethane for Adaptive Impact‐Resistant Protection with Robustness, Self‐Healing, and Recyclability

**DOI:** 10.1002/advs.202524271

**Published:** 2026-02-10

**Authors:** Rou‐Han Lai, Chia‐An Chiu, Yi‐An Chen, Athis Watwiangkham, Yu‐Hung Cheng, Yan‐Heng Chen, Min‐Han Yu, Lung‐Yi Lu, Chun‐Hsien Chen, Yi‐Ting Chen, Wei‐Hsiang Liao, Shang‐Hsiu Hu, Hsin‐Lung Chen, Siriporn Jungsuttiwong, Ho‐Hsiu Chou

**Affiliations:** ^1^ Department of Chemical Engineering National Tsing Hua University Hsinchu Taiwan; ^2^ Department of Chemistry and Center of Excellence for Innovation in Chemistry Faculty of Science Ubon Ratchathani University Ubon Ratchathani Thailand; ^3^ College of Semiconductor Research National Tsing Hua University Hsinchu Taiwan; ^4^ Department of Biomedical Engineering and Environmental Sciences National Tsing Hua University Hsinchu Taiwan; ^5^ Department of Chemical Engineering Faculty of Engineering Chulalongkorn University Bangkok Thailand

**Keywords:** *π*–*π* stacking interactions, hierarchical hydrogen bonding, impact‐resistant elastomer, recyclable elastomer, self‐healing materials, supramolecular polyurethane

## Abstract

Conventional ceramic and metallic impact‐protective materials are strong yet brittle and heavy, whereas soft materials such as Sylgard 184 and Styrofoam provide limited energy absorption and structural resilience. Achieving high impact resistance together with intrinsic self‐healing and recyclability remains a long‐standing challenge for polymeric systems, as most high‐strength soft elastomers rely on permanent covalent networks that hinder their reprocessability. Taking inspiration from human articular cartilage—a natural impact‐dissipative yet non‐healable tissue—we developed a supramolecular polyurethane–urea elastomer (PU‐BAMB) that emulates its fibrous–matrix architecture by integrating hierarchical hydrogen bonding and *π*–*π* stacking interactions through an aromatic diamine chain extender. This molecular design reproduces the multilevel energy‐dissipation mechanism of cartilage while overcoming its biological limitation by introducing intrinsic self‐healing and recyclability. The cooperative supramolecular framework achieves a finely tuned synergy between elasticity and rigidity, resulting in remarkable tensile strength (21.08 MPa), high fracture energy (138.36 kJ m^−^^2^), and rapid self‐healing (97% recovery within 1 h at 90°C). PU‐BAMB exhibits pronounced hysteresis, strain‐rate‐induced stiffening, and outstanding impact‐mitigation efficiency while retaining lightweight flexibility and sustainability. This work establishes a bio‐inspired yet functionally advanced design paradigm for constructing robust, self‐healing, and recyclable impact‐resistant elastomers for next‐generation protective coatings, damping systems, and adaptive wearable devices.

## Graphical abstract

Cartilage‐inspired supramolecular polyurethane–urea (PU‐BAMB) elastomers are developed by integrating hierarchical hydrogen bonding and *π*–*π* stacking interactions within a dynamically reconfigurable network. This synergistic supramolecular architecture overcomes the intrinsic trade‐off between rigidity and recoverability, imparting exceptional toughness, rapid self‐healing, and outstanding impact resistance, enabling PU‐BAMB as a lightweight and recyclable impact‐protection material.

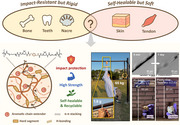

## 1 Introduction

Impact‐resistant materials play a crucial role in diverse fields such as personal protection, robotics, and aerospace structures [1]. These materials must endure sudden, high‐energy impacts while effectively dissipating mechanical energy to prevent catastrophic failure. Conventional systems based on rigid materials such as metals [2], ceramics [3, 4], and high‐modulus composites [5, 6] exhibit high stiffness and strength but suffer from brittleness, heaviness, and poor adaptability. Their limited deformability and irreversible damage under repeated impacts greatly restrict their use in flexible and adaptive protection systems such as wearable protection [7, 8], soft robotics [9, 10, 11], and adaptive architectures [12, 13]. In response, soft polymeric and supramolecular materials have emerged as promising alternatives, enabling molecular‐level control of elasticity, energy dissipation, and structural resilience through rational molecular design.

Among these soft systems, polyurethane (PU) elastomers stand out as particularly attractive candidates for impact‐protective applications owing to their lightweight nature, mechanical flexibility, and highly tunable phase‐segregated architecture [14, 15, 16, 17, 18]. By tailoring the balance between soft and hard segments, as well as modulating the hydrogen‐bonding density and crosslinking degree, PU networks can deliver programmable energy absorption and dynamic damping behavior under high strain rates [17, 19, 20]. In parallel, a wide range of high‐toughness polyurethane elastomers have been developed through rational hard‐segment design [21, 22], enhanced hydrogen‐bonding density [23, 24, 25, 26], and microphase‐segregation engineering [27, 28, 29]. These strategies have proven highly effective in achieving excellent tensile toughness and stretchability. Building upon these established approaches, alternative design pathways that emphasize dynamic and hierarchical network organization offer additional opportunities to further enrich mechanical functionality. In particular, introducing multiple reversible interactions across different length scales enables not only mechanical reinforcement but also adaptive energy dissipation, autonomous recovery, and improved sustainability, which are highly desirable for impact‐protective and reusable soft materials.

To further enhance impact resistance ability, supramolecular polymer networks (SPNs) have recently gained attention as a powerful molecular design platform for energy‐dissipative soft materials. By introducing dynamic and reversible interactions—such as multi‐strength hydrogen bonds [30, 31, 32, 33], ionic coordination [34, 35], and reversible covalent chemistry [36, 37, 38]—SPNs can reversibly reorganize their network structure under impact, achieving high strength and strain‐rate‐dependent stiffening. Representative studies include the poly(ionic liquid) double‐network elastomer reported by Li et al., which exhibited exceptional impact resistance and damping performance by coupling dynamic ionic coordination with a robust covalent framework, enabling rapid energy recovery under high strain rates [39]. Likewise, Qiao et al. also designed an entropy‐driven supramolecular network featuring highly specific guanidinium–carboxylate salt‐bridge hydrogen bonds, enabling a reversible soft‐to‐rigid transition with an ultrahigh impact‐stiffening ratio [40]. However, despite their outstanding impact resistance performance, many of these systems are still constructed o**n** permanent rigid covalent backbones, where the supramolecular motifs serve only as secondary dynamic components. As a result, the overall networks remain non‐recyclable and incapable of self‐healing, fundamentally limiting their sustainability and long‐term applicability in practical protection systems. Therefor**e,** developing a recyclable and self‐healable supramolecular elastomer that maintains high impact resistance without compromising dynamic recoverability remains a critical and unresolved challenge in the field of soft impact‐protection materials.

**FIGURE 1:**
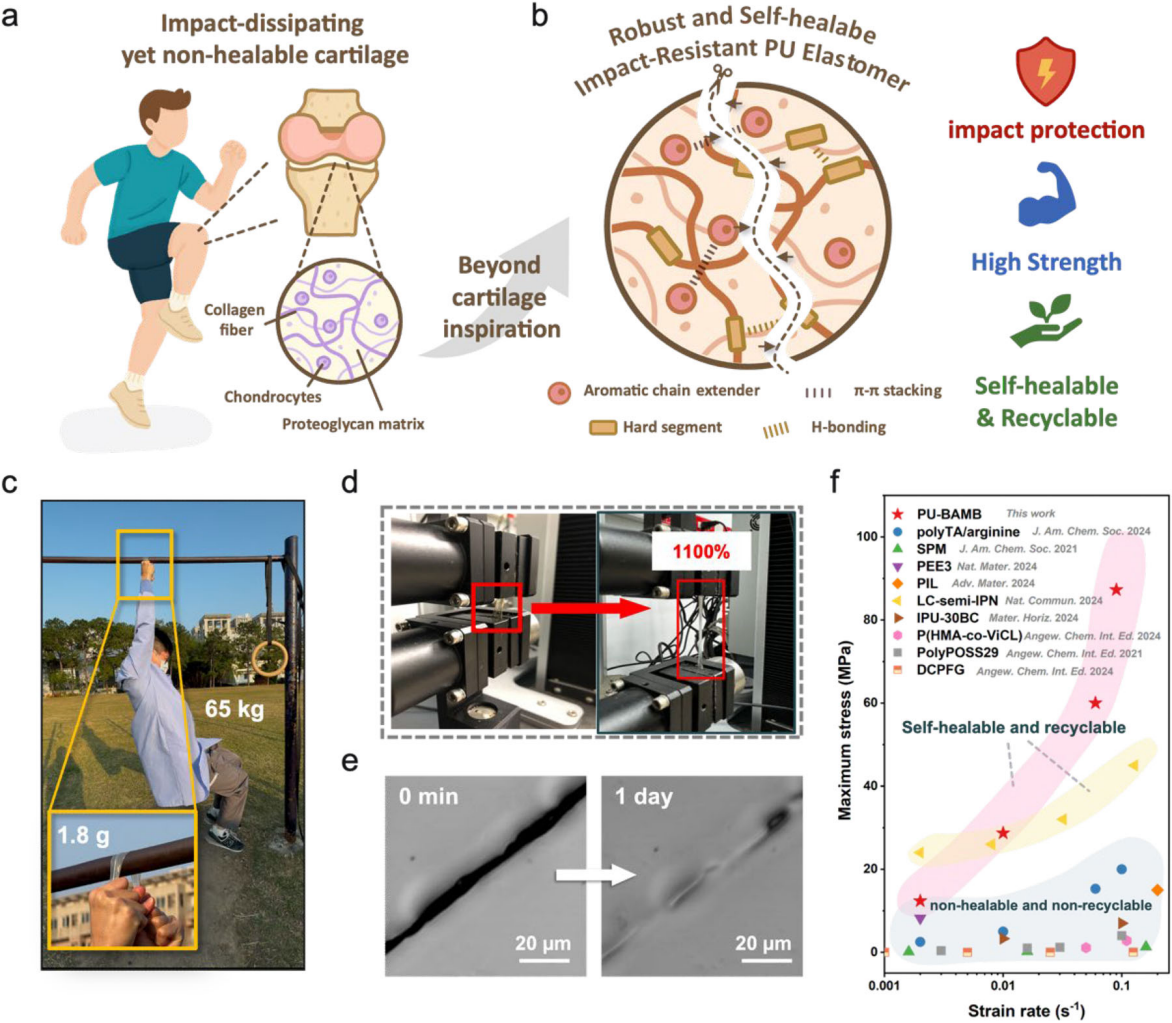
Design of the multifunctional PU‐BAMB elastomer. (a) Schematic illustration of the structure of articular cartilage. (b) Design strategy of PU‐BAMB mimicking the cartilage‐inspired rigid–flexible architecture through *π*–*π* stacking and hierarchical hydrogen‐bonding interactions. (c) Digital photograph showing that a 1.8 g PU‐BAMB film can support the weight of a 65 kg adult. (d) Digital photograph illustrating the high stretchability (∼1100%) of PU‐BAMB. (e) Confocal microscopy image showing the self‐healing ability of PU‐BAMB after 24 h at room temperature. (f) Comparison of the PU‐BAMB elastomer in this work with previously reported impact‐resistant materials in terms of compressive stress and strain rate [33, 38, 39, 40, 41, 42, 43, 44, 45].

To achieve this goal, we drew inspiration from biological tissues that intrinsically couple strength with efficient energy dissipation. Articular cartilage serves as a representative example because its hierarchically organized fibrous–matrix architecture enables it to absorb and dissipate the intense impact stresses generated during dynamic motions such as jumping and landing (Figure 1a) [46]. However, despite its exceptional load‐bearing capability, human articular cartilage exhibits extremely limited self‐healing due to its avascular nature. This biological limitation inspired us to create a synthetic counterpart that not only reproduces its impact‐dissipative architecture but also achieves autonomous self‐healing and recyclability. Inspired by this biological architecture, we developed a supramolecular polyurethane‐urea elastomer (PU‐BAMB) that mimics the synergistic coupling between fibrous reinforcement and elastic matrix through a rational rigid–flexible network design. In this design, poly(tetramethylene ether glycol) (PTMEG) and polyethylene glycol (PEG) were selected as the soft segments to emulate the hydrated matrix. The semi‐crystalline PTMEG provides molecular alignment and chain regularity, promoting elastic recovery and strain‐induced crystallization under deformation [47], while PEG, known for its excellent biocompatibility and hydrophilicity, enhances interchain mobility and contributes to a flexible, reversible network structure [48]. These soft segments impart high elasticity and energy absorption capability to the polymer backbone. Meanwhile, the hard domains constructed from urea/urethane linkages and the aromatic chain extender p‐xylylenediamine (BAMB) emulate the collagen fiber framework. The aromatic BAMB units generate *π*–*π* stacking interactions within the hard domains, which, in concert with strong and weak hydrogen bonds, form a hierarchical supramolecular network that facilitates microphase separation and promotes efficient energy dissipation under stress (Figure 1b). As a result, the PU‐BAMB elastomer exhibits exceptional mechanical robustness, with a lightweight 1.8 g film capable of supporting the hanging weight of an adult (≈65 kg)—approximately 360 000 times its own weight—highlighting its outstanding toughness and structural integrity (Figure 1c). Moreover, the material demonstrates remarkable stretchability, sustaining a strain of up to 1100% without fracture (Figure 1d). Confocal microscopy further confirms its intrinsic self‐healing capability, where surface scratches show pronounced autonomous healing, indicating autonomous self‐healing behavior of the material (Figure 1e). Benefiting from its hierarchical supramolecular architecture, PU‐BAMB achieves superior compressive performance across various strain rates, surpassing most reported soft impact‐resistant systems that, despite their high stiffness, lack intrinsic self‐healing and recyclability (Figure 1f).

## 2 Results and Discussion

### 2.1 Supramolecular Polyurethane (PU‐X) Elastomer Preparation and Characterization

**FIGURE 2:**
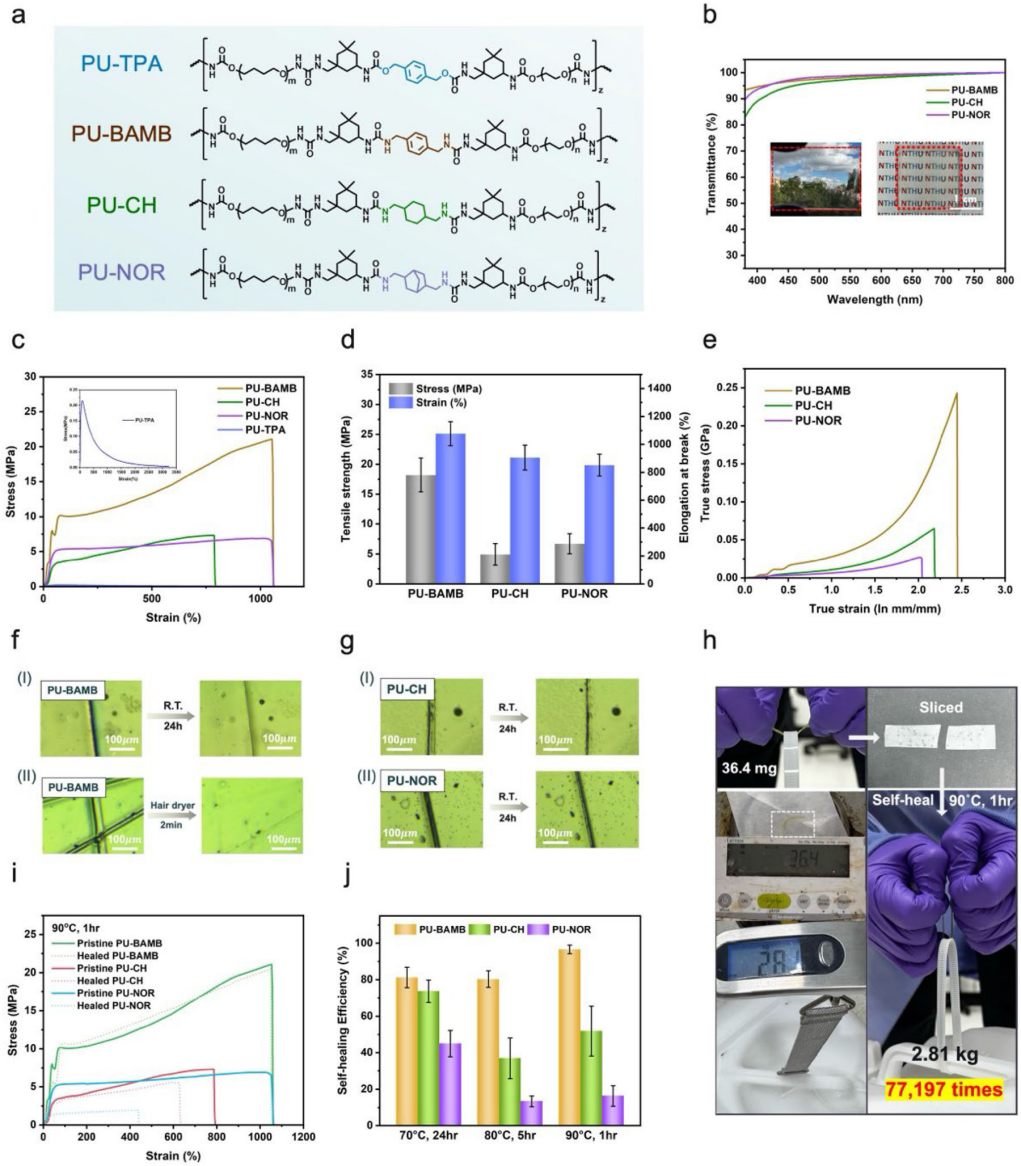
(a) Structural comparison among PU‐TPA, PU‐BAMB, PU‐CH, and PU‐NOR. (b) UV–vis spectra (350–800 nm) and digital photograph of PU‐BAMB (thickness ≈ 0.5 mm; 3 cm × 3 cm). (c) Stress–strain curves of PU‐BAMB, PU‐CH, and PU‐NOR. (d) Comparison of tensile strength and elongation for the series of elastomers. (e) Typical true stress–strain curves of PU‐BAMB, PU‐CH, and PU‐NOR. (f) Optical micrographs showing the scratch recovery behavior of PU‐BAMB at room temperature and after hair‐dryer treatment. (g) Optical micrographs of scratch tests for PU‐CH and PU‐NOR at room temperature. (h) A healed PU‐BAMB sample supporting a load exceeding 77 000 times its own weight. (i) Stress–strain curves of pristine and healed elastomers after healing at 90°C for 1 h. (j) Self‐healing efficiency of fractured elastomers under various healing conditions.

Building upon the cartilage‐inspired design concept, a series of supramolecular polyurethane (PU‐X) elastomers were synthesized to validate the proposed rigid–flexible coupling strategy and elucidate the structure–property relationships governing mechanical reinforcement and self‐healing behavior. In this design, the soft segment consisted of long‐chain polytetramethylene ether glycol (PTMEG), which provides high chain mobility and enables strain‐induced crystallization, combined with polyethylene glycol (PEG) to enhance biocompatibility. PTMEG and PEG were mixed at a molar ratio of 7:3, an optimized composition that balances mechanical strength and flexibility. For the hard segment, isophorone diisocyanate (IPDI) was selected as the diisocyanate component due to its asymmetric cyclic structure, which imparts rigidity while maintaining a degree of flexibility. To further build up the hierarchical supramolecular network, chain extenders with distinct structural features—1,4‐benzenedimethanol (TPA), p‐xylylenediamine (BAMB), 1,4‐cyclohexanebis(methylamine) (CH), and bis(aminomethyl)norbornane (NOR)—were employed to systematically study the effects of aromaticity and steric hindrance on the PU network (Figure 2a). These elastomers were denoted as PU‐X, where X represents TPA, BAMB, CH, or NOR, respectively.

The design and synthesis process of the PU‐X series polymers are shown in Figure S1. The terminal hydroxyl or amine groups of the chain extenders reacted with the isocyanate‐terminated prepolymer to form urethane or urea linkages, yielding linear polyurethane–urea elastomers. The chain extender content was systematically varied to optimize the coordination density within the polymer matrix, with the molar ratio relative to the soft segments adjusted from 0.5 to 2.0. For PU‐BAMB elastomers, ratios above 1.0 led to aggregation of excess BAMB, resulting in inhomogeneous films (Figure S2). Furthermore, increasing the BAMB content shifted the mechanical behavior from viscoelastic to hard and brittle (Figure S3 and Table S1), along with a decrease in self‐healing capability, as shown in Figure S4. Based on these findings, a molar ratio of 1.0 was selected as the optimal composition, providing a balance between mechanical performance and self‐healing efficiency. The same 1.0 molar ratio was applied for all other chain extenders (CH, TPA, and NOR) to enable direct comparison of their effects.

First, the progress of polymerization was tracked using Fourier transform infrared spectroscopy (FT‐IR). After the reaction between BAMB and the isocyanate‐terminated prepolymer, the characteristic –NCO stretching band at 2275 cm^−^^1^ completely disappeared, indicating full consumption of the isocyanate groups and confirming the completion of the condensation reaction (Figure S5). Furthermore, Gel Permeation Chromatography (GPC) analysis confirmed the increase in number‐average molecular weight (M_n_), demonstrating the successful chain extension and polymerization of the elastomers (Table S2). Thermogravimetric analysis (TGA) showed that all elastomers (PU‐BAMB, PU‐CH, and PU‐NOR) exhibited decomposition temperatures (T_d_) above 250°C, indicating good thermal stability (Figure S6 and Table S3). Two main weight‐loss stages were observed: the first between 250°C and 350°C, attributed to bond cleavage and decomposition in the soft segment (∼50% weight loss), and the second between 350°C and 450°C, associated with degradation of the hard segment (∼50% weight loss) [49]. Dynamic mechanical analysis (DMA) was conducted to determine the glass transition temperatures (T_g_) of all elastomers. The T_g_ around −80°C indicates that the polymers remain in a rubbery state at room temperature, ensuring adequate chain mobility for self‐healing (Figure S7 and Table S4). Variable‐temperature proton nuclear magnetic resonance (VT ^1^H‐NMR) further confirmed the dynamic nature of hydrogen bonding in the polymer network (Figure S8). For PU‐BAMB, the N─H protons in the urea groups appeared at 6.94 ppm at 30°C and progressively shifted upfield and broadened with increasing temperature, indicating hydrogen bond dissociation [47, 50]. Complementary variable‐temperature FT‐IR measurements showed blueshifts in both free and hydrogen‐bonded C═O stretching bands upon heating, suggesting that elevated temperatures promote hydrogen bond dissociation, chain rearrangement, and interdiffusion (Figure S9) [51]. These results confirmed that even densely packed hydrogen bonds in the hard domains remain dynamic at ambient temperature, enabling self‐healing. The optical transparency of the elastomer films was characterized by UV–vis spectroscopy. As shown in Figure 2b, the self‐supporting films of PU‐BAMB, PU‐CH, and PU‐NOR exhibited high optical transmittance of approximately 95% at 550 nm, confirming their excellent clarity and suggesting strong potential for applications in optically transparent or flexible electronic materials.

### 2.2 Supramolecular Polyurethane (PU‐X) Elastomer Mechanical Properties and Self‐Healing Performance

The mechanical properties of the elastomers were evaluated by tensile testing at room temperature with a stretching rate of 100 mm min^−^^1^. Among the series, PU‐BAMB, a urea‐linked elastomer with an aromatic backbone, displayed the best balance of strength and ductility. It reached a tensile strength of 21.08 MPa and an elongation at break of about 1056%. PU‐CH, which contains an alicyclic structure with urea linkage, exhibited a tensile strength of 7.31 MPa and an elongation of 790%, while PU‐NOR, with a bicyclic backbone, showed 3.59 MPa and 732%, respectively (Figure 2c; Table S5). In contrast, PU‐TPA, which is an aromatic urethane‐linked elastomer, possessed an ultrahigh elongation of 3230% but only 0.21 MPa in tensile strength (Figure S10). The material was too soft to maintain a free‐standing form, and therefore it was not subjected to further detailed studies. These results indicate that the chemistry of the hard segment strongly influences the overall mechanical response, and PU‐BAMB clearly outperformed the other systems (Figure 2d). PU‐BAMB elastomer also showed a characteristic nonlinear stress–strain behavior that can be divided into three distinct regimes (Figure S11). In the low‐strain region (0%–80%, Region I), the initial modulus was determined mainly by the density of hydrogen‐bonding crosslinks. When the strain increased to the intermediate region (80%–600%, Region II), the slope of the stress–strain curve decreased because weaker hydrogen bonds gradually dissociated, allowing efficient energy dissipation. At high strains (600%–1056%, Region III), the material exhibited pronounced strain‐hardening, which originated from the resistance of the permanent chain network [52, 53]. This multistage deformation behavior is similar to that of natural skin and reflects the synergistic effect of the hierarchical supramolecular network, which provides PU‐BAMB with remarkable toughness. A comparison between PU‐TPA and PU‐BAMB further illustrates the role of hydrogen bonding in the polymer system. PU‐TPA contained only urethane–urethane interactions, while PU‐BAMB benefited from stronger urea–urea bonds that created higher energy barriers and improved mechanical robustness [54]. Density functional theory (DFT) calculations using the B3LYP/6‐31g + DFT/D3(BJ) method also supported this conclusion. As shown in Figure S12, the binding energy of urea–urea hydrogen bonds was higher than that of urethane–urethane bonds. As a result, PU‐TPA lacked both mechanical strength and self‐supporting ability, whereas PU‐BAMB, PU‐CH, and PU‐NOR, which combined urea–urea and urethane–urea bonds, exhibited superior tensile strength and structural stability. The differences in mechanical properties among PU‐BAMB, PU‐CH, and PU‐NOR can be attributed to the distinct chain packing induced by their respective chain extenders. Dynamic mechanical analysis provided additional evidence (Figure S13a). The storage modulus (E′) of all three elastomers decreased continuously with increasing temperature, which was consistent with the rapid dissociation of dynamic hydrogen bonds. And PU‐BAMB consistently maintained the highest modulus throughout the temperature range. When the BAMB content increased, the decrease of E′ became progressively slower (Figure S13b). This effect was attributed to the stabilizing contribution of *π*–*π* stacking within the PU‐BAMB network, confirming the dominant role of *π*–*π* interactions in reinforcing the polymer matrix. In addition to its tensile strength, PU‐BAMB exhibited excellent fracture toughness. Notched PU‐BAMB samples could be stretched to five times their original length without catastrophic failure, and the fracture energy reached 138.36 kJ m^−^^2^, which is much higher than that of conventional self‐healing polyurethane elastomers (Figure S14 and Table S6). True stress–strain analysis provided a more reliable measure of load‐bearing capacity than engineering curves, because the cross‐sectional area decreased sharply near fracture [55, 56]. As shown in Figure 2e, the true stress of PU‐BAMB reached 0.25 GPa, far higher than that of the other systems.

The self‐healing performance of PU‐BAMB, PU‐CH, and PU‐NOR was systematically evaluated to elucidate how molecular architecture influences dynamic network recovery. Optical microscopy scratch‐healing tests revealed that PU‐BAMB exhibited the most efficient healing behavior. Visible crack closure occurred within one day at room temperature, and nearly complete disappearance was achieved within 2 min under mild heating with a household hair dryer (Figure 2f,g). Quantitative healing efficiency, defined as the ratio of the toughness of the healed sample to that of the pristine one, was further measured under three thermal conditions (70°C for 24 h, 80°C for 5 h, and 90°C for 1 h) (Figure S15 and Table S7). Under all conditions, PU‐BAMB consistently exhibited the highest recovery efficiency among the series (Figure 2i,j). At 90°C, PU‐BAMB demonstrated particularly rapid recovery, regaining a tensile strength of 20.6 MPa within 10 min, which corresponded to 97% of its original value (Figure S16 and Table S8). A striking demonstration of this capability is shown in Figure 2h, where a healed PU‐BAMB strip weighing only 36.4 mg (3.93 mm wide, 0.32 mm thick) successfully lifted a 2.8 kg weight, approximately 77 000 times its own weight, without interfacial failure and providing clear visual evidence of its robust self‐healing performance.

In addition to its intrinsic self‐healing ability, PU‐BAMB also exhibited excellent recyclability, which arises from its dynamic and reconfigurable supramolecular network, through a solvent‐assisted regeneration process. Damaged films were cut into small fragments, re‐dissolved in THF, and re‐cast onto PTFE molds to form new films (Figure S17a). The regenerated PU‐BAMB maintained nearly identical mechanical properties to the pristine samples even after three recycling cycles (Figure S17b), confirming the outstanding stability and reprocessability of its supramolecular framework. Taken together, the combination of dense reversible hydrogen bonding, efficient self‐healing, and recyclability significantly extends the service lifetime of PU‐BAMB while reducing material waste and cost. These features, along with its excellent mechanical robustness, underscore its promise as a sustainable and high‐performance material for impact‐resistant and protective applications.

### 2.3 Supramolecular Polyurethane (PU‐X) Elastomer Mechanical Properties and Self‐Healing Performance Mechanism

To elucidate the molecular origins underlying the remarkable mechanical strength and rapid self‐healing capability of PU‐BAMB, we systematically examined the supramolecular interactions and hierarchical structures across the PU‐X elastomer series. The distinct mechanical behaviors of PU‐BAMB, PU‐CH, and PU‐NOR arise primarily from differences in chain packing efficiency and intermolecular association governed by their respective chain extenders. Owing to its relatively small steric hindrance, PU‐BAMB enables tighter chain packing within the hard domains and facilitates the formation of an exceptionally dense hydrogen‐bonding network, allowing dynamic bonds to rupture and reform more effectively during the healing process. In contrast, the bulkier alicyclic and bridged‐bicyclic structures of PU‐CH and PU‐NOR restrict chain mobility and diminish the number of reconfigurable hydrogen‐bonding sites, resulting in slower chain rearrangement and reduced healing efficiency (Figure S18).

**FIGURE 3:**
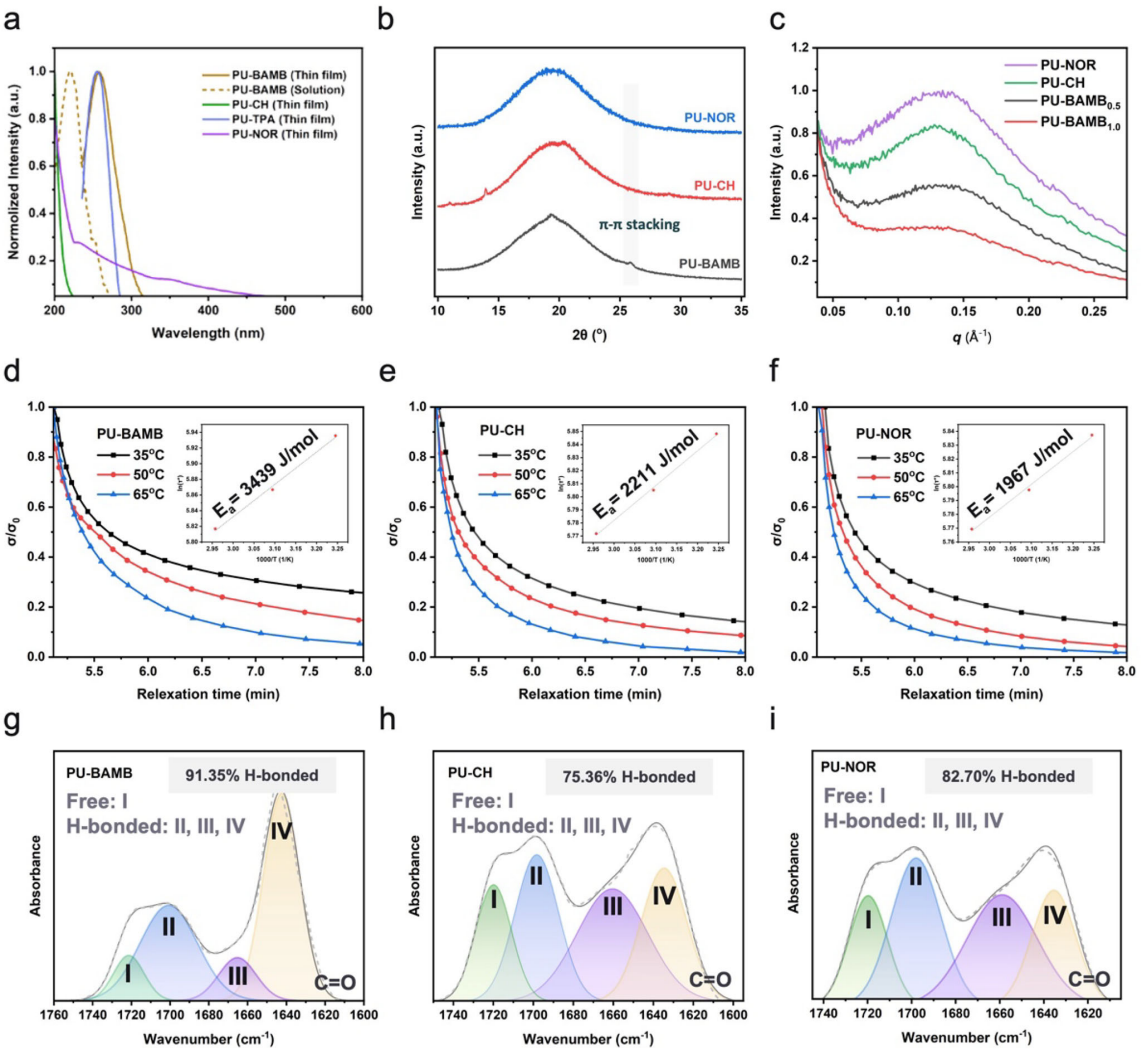
(a) UV–vis spectra of PU‐TPA, PU‐BAMB, PU‐CH, and PU‐NOR. (b) XRD patterns of PU‐BAMB, PU‐CH, and PU‐NOR. (c) SAXS profiles of PU‐BAMB_0.5_, PU‐BAMB_1.0_, PU‐CH, and PU‐NOR. Stress relaxation curves and calculated activation energies (E_a_) of (d) PU‐BAMB, (e) PU‐CH, and (f) PU‐NOR. Deconvoluted subpeaks of the FTIR C═O stretching vibration region for (g) PU‐BAMB, (h) PU‐CH, and (i) PU‐NOR.

Aromatic interactions were further identified as key contributors to PU‐BAMB's enhanced supramolecular cohesion. First, UV–vis spectroscopy provided direct evidence of such aromatic interactions in the polymer systems (Figure 3a). Both PU‐TPA and PU‐BAMB thin films exhibited absorption bands at around 260 nm, which are associated with *π*–π* transitions of aromatic rings [57]. PU‐BAMB displayed a noticeable redshift in the solid state compared with its solution, confirming that aromatic stacking became stronger and more compact in the condensed phase. In contrast, PU‐CH and PU‐NOR, which lack aromatic moieties, showed no such characteristic bands, and PU‐NOR exhibited a broader signal due to lower transparency. X‐ray diffraction (XRD) analysis further verified the presence of aromatic stacking in PU‐BAMB (Figure 3b). Although all samples were amorphous, PU‐BAMB displayed a weak but distinct diffraction signal attributed to *π*–*π* stacking [58]. The intensity of this peak increased with BAMB content, confirming its origin from the aromatic backbone (Figure S19). These dynamic interactions are expected to induce microphase separation and serve as supramolecular crosslinking sites, thereby enhancing both toughness and tensile strength. Small‐angle X‐ray scattering (SAXS) measurements also revealed distinct scattering peaks for PU‐NOR, PU‐CH, and PU‐BAMB (Figure 3c). The corresponding domain spacings, calculated from Bragg's equation, were 4.76 nm for PU‐NOR, 4.83 nm for PU‐CH, and 4.55 nm for PU‐BAMB. The smaller spacing of PU‐BAMB indicates tighter chain packing driven by aromatic stacking, whereas the bulkier PU‐NOR and PU‐CH formed more loosely organized domains. Increasing the BAMB content further reduced the spacing to 4.48 nm, providing additional confirmation of the *π*–*π* stacking effect. Thus, a moderate reduction in phase‐separated domain size, promoted by aromatic interactions, leads to higher tensile strength and elongation, while also facilitating chain rearrangement that improves self‐healing efficiency [47, 59]. To further visualize the nanoscale morphology, AFM phase imaging was conducted on PU‐NOR, PU‐CH, and PU‐BAMB (Figure S20). The AFM phase images provide contrast arising from variations in local viscoelastic responses, which are commonly associated with microphase‐separated structures in segmented polyurethane systems. In the phase images, the bright and dark regions reflect differences in local viscoelastic responses and segmental mobility within the polymer network. These observations offer direct morphological support for the presence of microphase separation and are consistent with the domain organization inferred from the SAXS results. Dynamic mechanical analysis (DMA) stress‐relaxation experiments further revealed the influence of chain extender chemistry on segmental dynamics (Figure 3d–f). At a fixed strain of 0.01%, the characteristic relaxation time (τ*) was defined as the time required for the modulus to decay to 1/e of its initial value. The calculated activation energies (*E_a_*) differed significantly among the samples. PU‐BAMB exhibited the highest value of 3439 J mol^−^^1^, reflecting stronger intermolecular interactions and a more stable network structure, consistent with its superior storage modulus and mechanical strength [60]. By comparison, PU‐NOR and PU‐CH displayed lower activation energies of 2211 and 1967 J mol^−^^1^, respectively, correlating with their weaker mechanical properties. To gain deeper insight into the hydrogen‐bonding structures within the elastomers, FT‐IR spectroscopy was conducted to analyze the carbonyl stretching region (1600–1750 cm^−^^1^). Peak deconvolution revealed four distinct components: (I) free carbonyls, (II) ordered urethane–urethane hydrogen bonds, (III) disordered urea–urea hydrogen bonds, and (IV) ordered urea–urea hydrogen bonds (Figure S21) [61, 62]. As shown in Figure 3g–i, the proportion of hydrogen‐bonded carbonyls increased significantly from 75.36% in PU‐CH and 82.70% in PU‐NOR to 91.35% in PU‐BAMB, with the most pronounced enhancement arising from the ordered urea–urea hydrogen bonds (peak IV). This observation was further supported by quantitative results summarized in Table S9. Taken together, these findings demonstrate that the cooperative interplay between ordered hydrogen bonding and *π*–*π* stacking establishes a robust and dynamic supramolecular framework within PU‐BAMB. This hierarchical interaction network not only enhances mechanical reinforcement but also provide chain rearrangement, endowing the elastomer with exceptional toughness, self‐healing capability, and structural stability.

### 2.4 Supramolecular Polyurethane (PU‐X) Elastomer Energy Dissipation Performance

**FIGURE 4:**
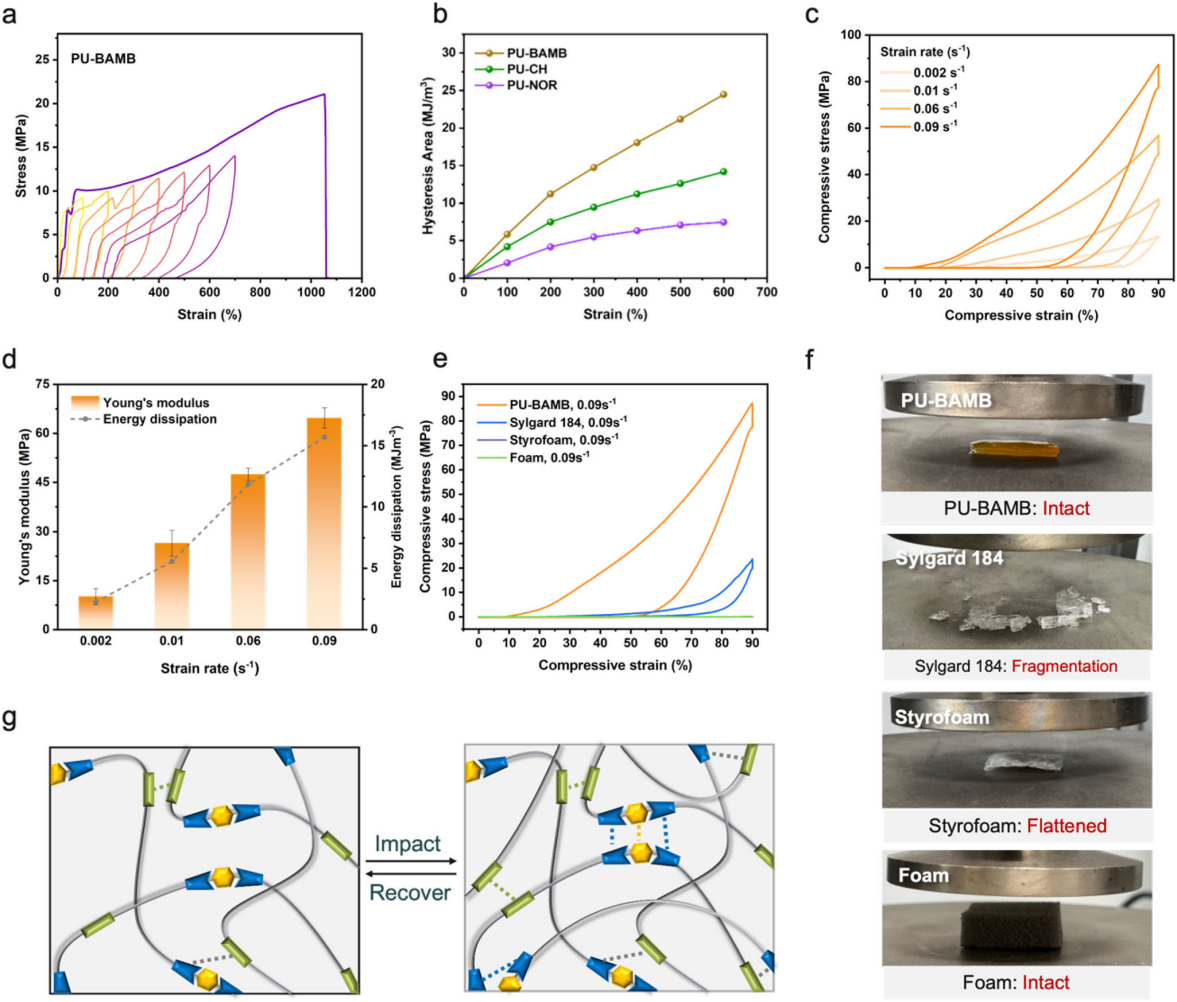
(a) Cyclic tensile stress–strain curves of PU‐BAMB under successive loading–unloading cycles at increasing strain levels (100%–600%) without resting intervals. (b) Comparison of hysteresis areas for PU‐BAMB, PU‐CH, and PU‐NOR. (c) Cyclic compression stress–strain curves of PU‐BAMB measured at various strain rates. (d) Young's modulus and energy dissipation of PU‐BAMB as a function of strain rate. (e) Cyclic compression performance of PU‐BAMB, Sylgard 184, Styrofoam, and sponge foam at a strain rate of 0.09 s^−^^1^. (f) Digital photographs of PU‐BAMB, Sylgard 184, Styrofoam, and sponge foam after compression testing. (g) Schematic illustration of the impact‐stiffening mechanism of PU‐BAMB, enabled by interfacial hydrogen bonding and *π*–*π* stacking interactions.

PU‐BAMB elastomers exhibited a pronounced hysteresis behavior during cyclic tensile tests, demonstrating excellent energy‐dissipation capability [63]. The multistep cyclic stress–strain experiments further revealed a strain‐dependent hysteretic response, where the loop area increased progressively with strain, indicating enhanced energy dissipation (Figure 4a) [64]. This behavior originates from a hierarchical supramolecular network formed by the BAMB chain extender, where multilevel hydrogen bonding and *π*–*π* interactions cooperatively restrict chain mobility and dissipate energy during deformation. As a result, PU‐BAMB displayed substantially greater hysteretic energy dissipation ability than PU‐CH and PU‐NOR elastomers (Figure 4b; Figure S22). To further corroborate this dynamic damping behavior, frequency‐sweep rheological tests were performed in the range of 0.1–70 Hz (Figure S23). PU‐BAMB exhibited stable storage (G′) and loss (G′) modulus across the entire frequency range, confirming the integrity of its supramolecular network [39]. The gradual increase of G′ with frequency reflects the enhanced elastic response of the dynamic network at higher deformation rates. Combined with the stress‐relaxation results showing that PU‐BAMB possesses the slowest relaxation dynamics and the highest activation energy among the series, these findings indicate that its supramolecular network resists rapid molecular rearrangement, thereby sustaining higher stress and promoting effective energy dissipation under fast deformation.

Given its remarkable stress‐dissipation ability under tension, the dynamic compressive performance of PU‐BAMB was further examined. Strain rate–dependent cyclic compression tests revealed clear reinforcement behavior: both Young's modulus and dissipated energy increased with strain rate (Figure 4c,d). This rate‐hardening effect implies that, under rapid loading, the internal network undergoes transient densification as reversible crosslinks reorganize and additional entanglements form, resulting in a temporarily reinforced structure that resists further compression [40]. Distinct from conventional physically crosslinked elastomers governed by a single population of reversible junctions, the present material features a hierarchical supramolecular network composed of interactions spanning a broad spectrum of binding energies and dynamic timescales [65]. Specifically, weaker hydrogen bonds reorganize and dissipate energy at the early stages of deformation, while stronger, more ordered hydrogen‐bonding domains are progressively engaged to restrict chain mobility at higher loading rates. At even higher strain rates, *π*–*π* stacking interactions within the hard domains further contribute to load bearing and network stabilization. This sequential and cooperative activation of multilevel interactions enables rate‐adaptive stiffening through distributed energy dissipation rather than abrupt rigidification or localized stress concentration [66]. This mechanism is schematically illustrated in Figure 4g, where the synergistic reorganization of hydrogen bonds and *π*–*π* stacking interactions dynamically strengthens the network, enhancing its load‐bearing capacity and impact resistance. When compared with conventional protective materials such as Sylgard 184 silicone rubber, Styrofoam, and sponge foam, PU‐BAMB demonstrated the highest compressive stress at equivalent strain rates (Figure 4e; Figure S24). After compression, PU‐BAMB and the sponge foam retained their structural integrity, whereas Sylgard 184 fractured and Styrofoam was completely crushed, losing its resilience (Figure 4f). Collectively, these results demonstrate the outstanding mechanical robustness and energy‐absorbing efficiency of PU‐BAMB, confirming its promise as a lightweight, durable material for advanced soft impact‐protection applications.

### 2.5 Impact Resistance Performance

**FIGURE 5:**
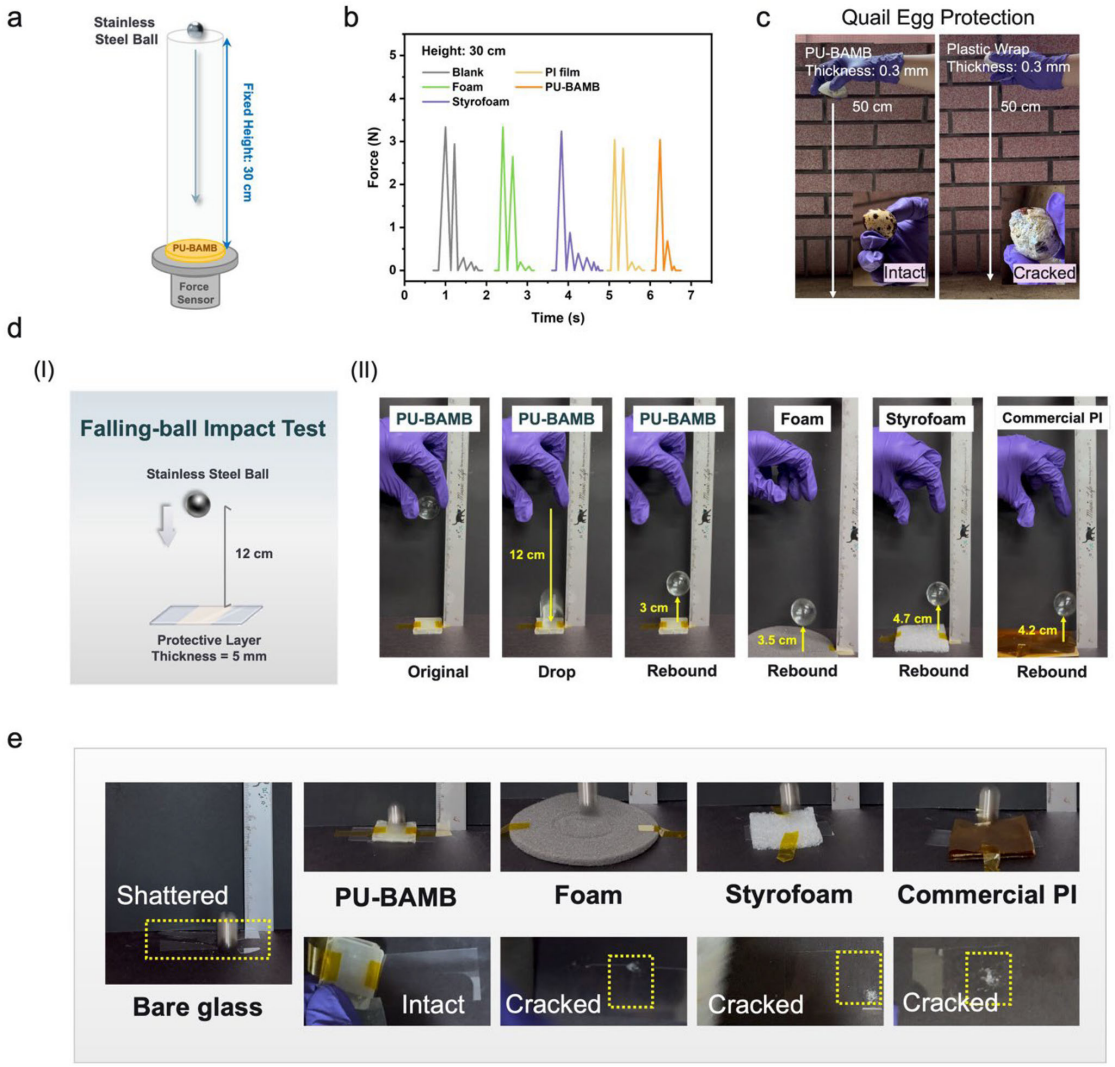
(a) Schematic illustration of the impact force attenuation test. (b) Time‐resolved impact force attenuation waves transmitted through the blank sample, foam, Styrofoam, PI film, and PU‐BAMB when a 20 g steel ball was dropped freely from a height of 30 cm. (c) Digital photograph of a quail egg wrapped with a PU‐BAMB elastomer (thickness ≈ 0.3 mm) and dropped from a height of 50 cm. The quail egg protected by PU‐BAMB remained intact, whereas the one wrapped with plastic film of the same thickness was cracked. (d) Falling‐ball impact test: (i) schematic diagram of the falling‐ball impact setup; (ii) comparative tests conducted on foam, Styrofoam, PI film, and PU‐BAMB. (e) Digital photographs of foam, Styrofoam, PI film, and PU‐BAMB (5 mm thickness) used as glass protection materials under impact conditions.

In addition, the energy absorption values obtained from the falling‐ball impact test clearly demonstrate the impact resistance of the materials. In this test, a force sensor was mounted beneath the samples to continuously monitor the variation of the transmitted force (Figure 5a). The results reveal that under a drop height of 30 cm, the PU‐BAMB elastomer was able to rapidly suppress the propagation of the impact wave, exhibiting markedly superior damping performance compared with the three control samples (foam, Styrofoam, and PI film) (Figure 5b). More specifically, PU‐BAMB effectively reduced the impact force transmitted to the substrate during the very first impact, achieving a rebound attenuation ratio of 77.4%, which is significantly higher than those of foam and PI film, thereby confirming its outstanding energy‐dissipation capability (Figure S25). It is noteworthy that although commercial Styrofoam exhibited a rebound attenuation ratio of 73%, its single‐use and non‐recyclable nature severely limits its practical applications. To further validate the protective capability of PU‐BAMB against impact, a quail egg drop test was conducted. When coated with a 0.3 mm‐thick PU‐BAMB elastomer layer, the quail egg remained intact and undamaged even after a free fall from a height of 50 cm (Figure 5c; Video S1). In contrast, when the egg was wrapped with multiple layers of commercial plastic wrap to achieve an equivalent thickness of 0.3 mm, it fractured upon impact under identical conditions, with visible cracks and leakage of egg fluid (Video S2). This striking difference highlights that PU‐BAMB can deliver remarkable impact protection even at a minimal thickness.

Furthermore, standardized falling‐ball impact tests were performed to quantitatively compare the energy absorption and protective performance of different materials. As illustrated in Figure 5d(i), a 19.08 g steel ball was dropped from a height of 0.12 m onto a 5 mm‐thick protective layer. The test results Figure 5d(ii) indicate that PU‐BAMB yielded the lowest rebound height of only ∼3 cm, much lower than those recorded for foam, Styrofoam, and PI film, demonstrating its superior capacity to dissipate the kinetic energy generated during impact. This finding further corroborates the excellent energy‐absorbing ability of PU‐BAMB.

Finally, to directly visualize the protective effect of energy dissipation, a glass protection experiment was designed. A 5 mm‐thick protective layer of each material was applied onto glass substrates, which were then subjected to the impact of a 19.08 g steel ball dropped from a height of 0.30 m. As shown in Figure 5e, among all tested materials, only the glass protected by PU‐BAMB remained completely intact without cracks, whereas foam‐, Styrofoam‐, and PI film‐protected glass samples all fractured. These results unambiguously highlight the superior protective and impact‐resistant properties of PU‐BAMB, underscoring its great potential as a high‐performance impact‐damping material.

## 3 Conclusion

In summary, we have developed a cartilage‐inspired supramolecular polyurethane–urea elastomer (PU‐BAMB) that integrates hierarchical hydrogen bonding and *π*–*π* stacking interactions through an aromatic diamine chain extender. This dynamic supramolecular framework enables a finely tuned synergy between elasticity and rigidity, imparting exceptional tensile strength (21.08 MPa), high fracture energy (138.36 kJ m^−^^2^), and rapid self‐healing efficiency (97% recovery within 1 h at 90°C). Drawing inspiration from the fibrous–matrix architecture of human articular cartilage, which dissipates impact energy efficiently but lacks intrinsic healing ability, PU‐BAMB not only emulates its hierarchical energy‐dissipation mechanism but also transcends this biological limitation through autonomous self‐healing and recyclability. Unlike conventional covalently crosslinked elastomers, PU‐BAMB achieves high impact resistance, dynamic self‐healing ability, and recyclability simultaneously, overcoming a long‐standing limitation in the design of soft protective materials. The reversible noncovalent interactions not only dissipate stress effectively under impact but also reorganize upon unloading, allowing repeated deformation without structural fatigue. This work establishes a sustainable supramolecular design strategy for constructing lightweight, recyclable, and adaptively tough elastomers that unite mechanical robustness, energy dissipation, and intrinsic self‐healing. Such materials offer compelling potential for next‐generation protective coatings, energy‐damping interfaces, and wearable impact‐resistant devices.

## Conflicts of Interest

The authors declare no conflicts of interest.

## Supporting information




**Supporting File 1**: advs74310‐sup‐0001‐SuppMat.docx.

**Supporting File 2**: advs74310‐sup‐0002‐VideoS1.mp4.

**Supporting File 3**: advs74310‐sup‐0003‐VideoS2.mp4.

## Data Availability

The data that support the findings of this study are available from the corresponding author upon reasonable request.
