# Supplementary material for: Beyond Cartilage‐Inspired Supramolecular Polyurethane for Adaptive Impact‐Resistant Protection with Robustness, Self‐Healing, and Recyclability
Source: Adv Sci (Weinh). 2026 Feb 10;13(22):e24271. doi: 10.1002/advs.202524271 (PMC13088313; doi:10.1002/advs.202524271)
Supplement: Supplementary file 1 — Supporting File 1: advs74310‐sup‐0001‐SuppMat.docx. [file ADVS-13-e24271-s003.docx]

**Beyond Cartilage-Inspired Supramolecular Polyurethane for Adaptive Impact-Resistant Protection with Robustness, Self-Healing, and Recyclability**

*Rou-Han Lai^a‡^, Chia-An* *Chiu^a‡^, Yi-An Chen^a^,* *Athis Watwiangkham^b^, Yu-Hung Cheng^a^, Yan-Heng* *Chen^a^,* *Min-Han* *Yu^a^,* *Lung-Yi Lu^a^, Chun-Hsien Chen^a^, Yi-Ting Chen^a^, Wei-Hsiang Liao^c^, Shang-Hsiu Hu^d^, Hsin-Lung Chen^a^, Siriporn Jungsuttiwong^b^,* *Ho-Hsiu Chou^a,c,e*^*

^a^Department of Chemical Engineering, National Tsing Hua University, Hsinchu 300044, Taiwan

^b^Department of Chemistry and Center of Excellence for Innovation in Chemistry, Faculty of Science, Ubon Ratchathani University, Ubon Ratchathani, 34190, Thailand

^c^College of Semiconductor Research, National Tsing Hua University, Hsinchu 300044, Taiwan

^d^Department of Biomedical Engineering and Environmental Sciences, National Tsing Hua University, Hsinchu 300044, Taiwan

^e^Department of Chemical Engineering, Faculty of Engineering, Chulalongkorn University, Bangkok 10330, Thailand

‡These authors contributed equally.
***These authors are the corresponding authors.

E-mail address: [hhchou@mx.nthu.edu.tw](mailto:hhchou@mx.nthu.edu.tw) (Ho-Hsiu Chou)

1. **Experimental Section**

## Materials

Polytetramethylene ether glycol (PTMEG, Mn = 650 g mol⁻¹), polyethylene glycol (PEG, Mn = 200 g mol⁻¹), and bis(aminomethyl)norbornane (NOR) were purchased from Tokyo Chemical Industry Co., Ltd. (TCI). Isophorone diisocyanate (IPDI, 99%), 1,4-benzenedimethanol (TPA), and p-xylylenediamine (BAMB) were obtained from Nova-Matls. Dibutyltin dilaurate (DBTDL, 95%) was supplied by Alfa Aesar, and 1,4-cyclohexanebis(methylamine) (CH) was purchased from Sigma-Aldrich. All chemicals were used as received without further purification.

## Synthesis of PU-X elastomer

All elastomers were synthesized via a two-step prepolymer method using commercially available raw materials. Taking the synthesis of PU-BAMB as an example: polyethylene glycol (PEG, M_n_ = 200; 0.3 mmol, 60 mg) and polytetramethylene ether glycol (PTMEG, M_n_ = 650; 0.7 mmol, 455 mg) were mixed in a 20 mL vial. The mixture was heated to 80 °C under vacuum for 1 h to remove residual moisture and oxygen, and then cooled to 60 °C. Subsequently, anhydrous THF (3 mL) was added to dissolve the soft segments under a nitrogen atmosphere. A solution of isophorone diisocyanate (IPDI, 2 mmol, 444.6 mg) in anhydrous THF (3 mL) together with dibutyltin dilaurate (DBTDL, 5 mg) was dropwise introduced and stirred at 60 °C for 2 h, affording an isocyanate-terminated prepolymer. For chain extension, a solution of p-xylylenediamine (BAMB, 1 mmol, 136.2 mg) in anhydrous THF (2 mL) was added dropwise into the prepolymer solution and stirred at 60 °C for 1 h to obtain PU-BAMB. PU-TPA, PU-CH, and PU-NOR were synthesized in the same method, replacing BAMB with the corresponding chain extender.

## Preparation of Free-Standing films

Reacted solutions were respectively poured into Teflon mold and evaporated the solvent at 50℃ to obtain free-standing films.

## Mechanical property test

The mechanical properties (uniaxial tensile tests and cyclic tensile tests) of all elastomer films (measuring 3 cm x 1 cm x 0.5 mm) were evaluated at room temperature using a uniaxial tensile tester with a displacement rate of 100 mm min^-1^. Each sample underwent testing with at least three specimens.

## Cyclic Compression Test

The cyclic compression tests were conducted at room temperature using a universal testing machine (QC-505M2F) equipped with a 40,000 N load cell. Rectangular specimens with dimensions of 30 mm × 10 mm × 2.5 mm were prepared. The tests involved single-cycle compression experiments with strain rates ranging from 0.002 s⁻¹ to 0.09 s⁻¹. The applied compressive strain was maintained below 100%, with detailed analyses conducted at a strain level of 90%.

## Self-Healing test

In the self-healing experiment, elastomer films underwent cutting into two pieces using a razor blade, followed by rejoining and healing in a 70, 80 and 90°C oven for different period of time (24, 5, and 1 h). Each sample underwent testing with at least three specimens. The self-healing efficiency is defined as follows:

$$Healing efficiency =\frac{{Toughness}_{healed}}{{Toughness}_{pristine}} \times100\%$$

## Recycling test

The elastomers were cut into small pieces using surgical scissors and redissolved in THF (HPLC grade) at a concentration of approximately 50 mg per mL. The solution was stirred at room temperature for 12 h to ensure complete dissolution, followed by ultrasonication to achieve uniform dispersion and remove air bubbles. The resulting solution was slowly poured into molds and left on a 50 °C heating plate for two days to obtain the films.

## Fracture energy test

Fracture energy was determined using both unnotched and notched specimens, the latter containing a single notch of 1 mm in length. All specimens had dimensions of 10.0 mm × 5.0 mm × 0.4 mm and were subjected to tensile testing at a constant crosshead speed of 100 mm min⁻¹. The fracture energy (G_c_) was calculated according to the following equation:

$\begin{matrix} & G_{c}=\frac{6wc}{\sqrt{\lambda_{c}}} & & \end{matrix}$

where c is the notch length (1 mm), λ_c_ is the elongation at break of the notched specimen, and w is the strain energy obtained by integrating the stress–strain curve of the unnotched specimen up to λ_c_.

## True Stress and True Strain

The true stress (σₜ) and true strain (εₜ) were calculated from the engineering stress–strain curves obtained at a tensile rate of 100 mm min⁻¹. The calculation formulas are as follows:

$$\text{True stress}\text{: }\sigma_{t}=\sigma\times\frac{L}{L_{0}}=\sigma(\varepsilon+1)$$

$$\text{True strain}\text{: }\varepsilon_{t}=\int_{L_{0}}^{L} \frac{dL}{L}=\ln\frac{L}{L_{0}}=\ln(\varepsilon+1)$$

where σ is the engineering stress, L is the instantaneous length of the deformed specimen, L₀ is the original gauge length, and ε is the engineering strain.

## Density Functional Theory (DFT) Calculations

Density functional theory (DFT) calculations were performed using the Gaussian 16 program^[1]^, at the B3LYP^[2]^/6-31G(d,p) level of theory, with the Grimme’s D3(BJ) dispersion correction^[3]^ to evaluate the hydrogen-bonding interactions between urea–urea, urethane–urea, and urethane–urethane dimers.

1.11 Impact force attenuation test

Impact force attenuation test was evaluated using a force sensor. A 5 mm thick sample (including commercial impact-resistant materials) was fixed onto the sensor, and a steel ball (20 g) was released from a fixed height (30 cm) through a transparent soft tube to ensure a consistent falling path. The impact response was recorded as force–time curves for subsequent analysis.

## 1.12 Falling Ball Impact Test

The samples were secured on a flat platform, with a ruler placed alongside as a height reference. A steel ball (19.08 g) was released from a fixed height of 0.12 m to impact the samples, each with a thickness of 5 mm, including commercial impact-resistant materials for comparison. The entire process was recorded using a high-speed camera, and the rebound height was analyzed based on the captured footage. In the glass protection test, the same setup and samples were used, and a steel ball weighing 19.08 g was dropped from a height of 0.3 m.

## Characterization

Fourier transform infrared (FT-IR) spectra were collected on a NICOLET iS50 FT-IR spectrometer in the range of 400–4000 cm⁻¹ with 32 scans at a resolution of 4 cm⁻¹.¹H nuclear magnetic resonance (NMR) spectra were recorded on a Bruker Avance 500 MHz spectrometer with samples dissolved in chloroform-d. Gel permeation chromatography (GPC) was performed on a Hitachi system with a 5450 RI detector calibrated with polystyrene standards, using tetrahydrofuran as the eluent at a flow rate of 1.00 mL min⁻¹. Thermogravimetric analysis (TGA) was carried out on a TA Q600 analyzer from 25 to 800 °C at a heating rate of 10 °C min⁻¹ under nitrogen. Optical microscopy (OM) images were obtained using a WHITED WM-100 system. UV–vis spectra were recorded on a Shimadzu UV-1900 spectrophotometer. Tensile tests were conducted using a QC-505M2F universal tester equipped with a 100 N load cell. Rectangular specimens (30 mm × 10 mm × 0.4 mm) were tested at a displacement rate of 100 mm min⁻¹. Dynamic mechanical analysis (DMA) was carried out on a TA Instruments Q800 analyzer in tension-film mode. The temperature range for T_g_ measurement was −140 to 60 °C with a heating rate of 3 °C min⁻¹ and a frequency of 1 Hz. X-ray diffraction (XRD) patterns were recorded on a Bruker D8 ADVANCE diffractometer (Germany). Confocal laser scanning microscopy (CLSM) images were acquired using a Carl Zeiss LSM800 confocal microscope, Oberkochen, Germany.

## Statistical Analysis and Data Reproducibility

All mechanical, self-healing, and impact-resistance measurements were conducted using at least three independent specimens unless otherwise stated. The reported values are presented as the mean ± standard deviation. Stress–strain curves shown in the figures are representative results, while the summarized data in the tables were obtained from statistical analysis of repeated measurements. This approach ensures the reliability and reproducibility of the experimental results.


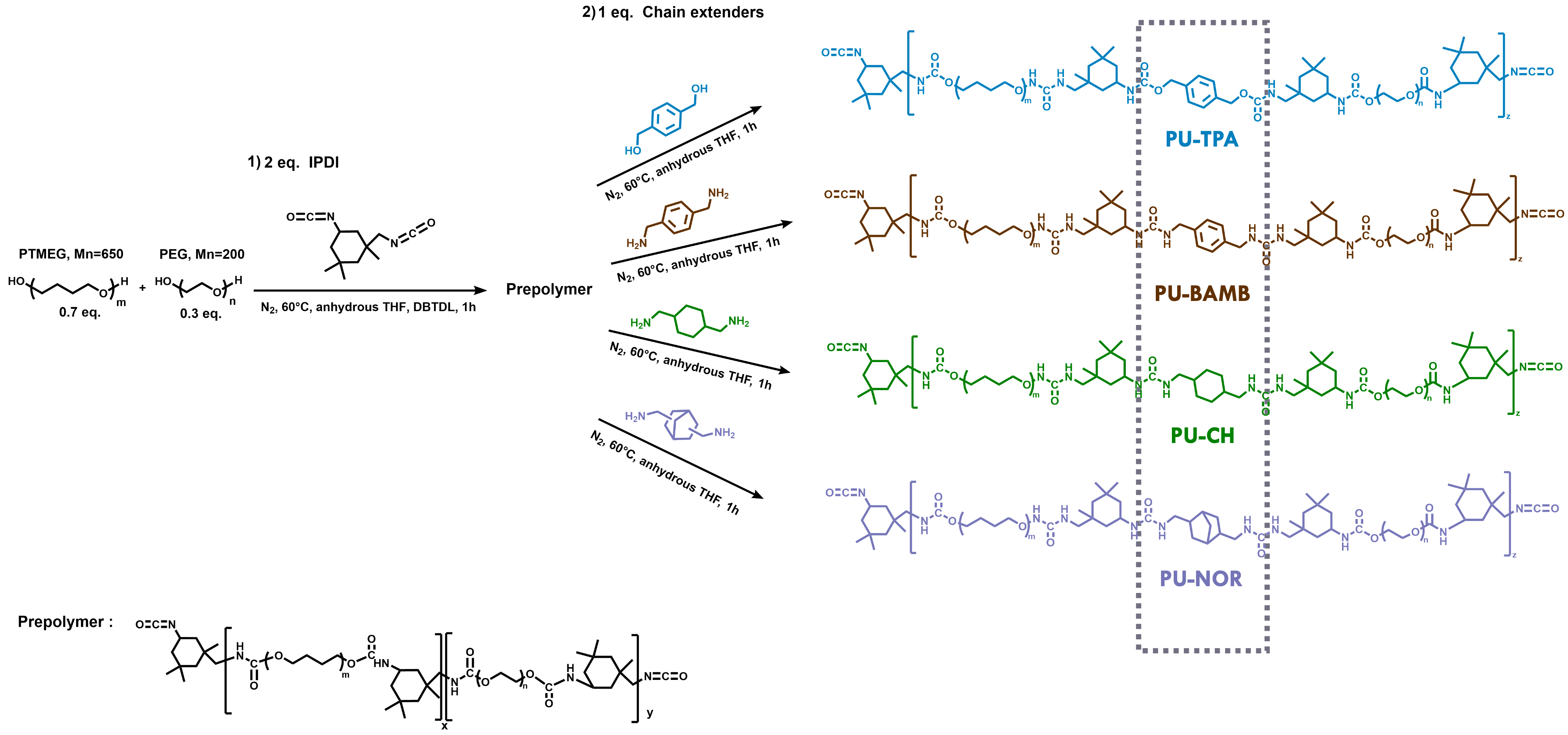


**Figure S1** Synthesis process of PU-X series polymer. (x=TPA, BAMB, CH, and NOR)


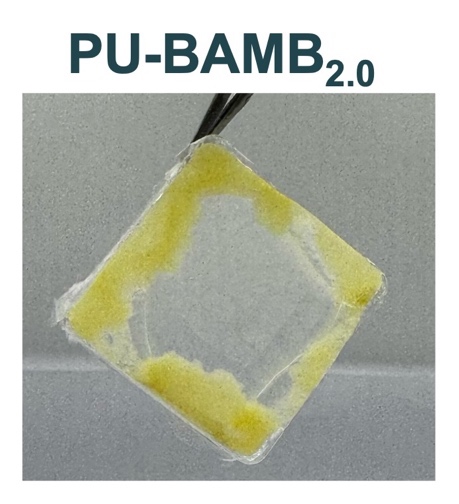


**Figure S2**. Digital photo of the non-uniform PU-BAMB_2.0_ film.


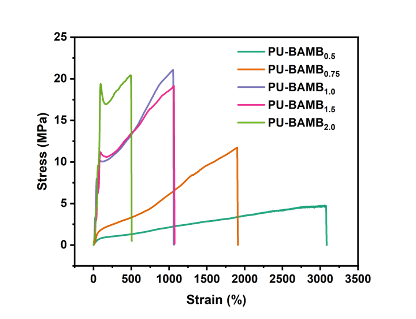


**Figure S3** Mechanical properties of elastomers with varying BAMB feed ratios.

**Table S1** Mechanical properties of elastomers of PU-BAMB_x_ (x = molar ratio relative to the soft segments)

| Ratio | Tensile Strength (MPa) | Elongation at break (%) | Modulus (MPa) | Toughness  (MJm^-3^) |
| --- | --- | --- | --- | --- |
| 0.5 | 4.77 | 3078 | 0.15 | 87.39 |
| 0.75 | 11.73 | 1902 | 1.59 | 116.93 |
| 1.0 | 21.08 | 1056 | 9.46 | 149.64 |
| 1.5 | 19.16 | 1068 | 11.93 | 145.68 |
| 2.0 | 20.43 | 500 | 21.55 | 82.47 |

**
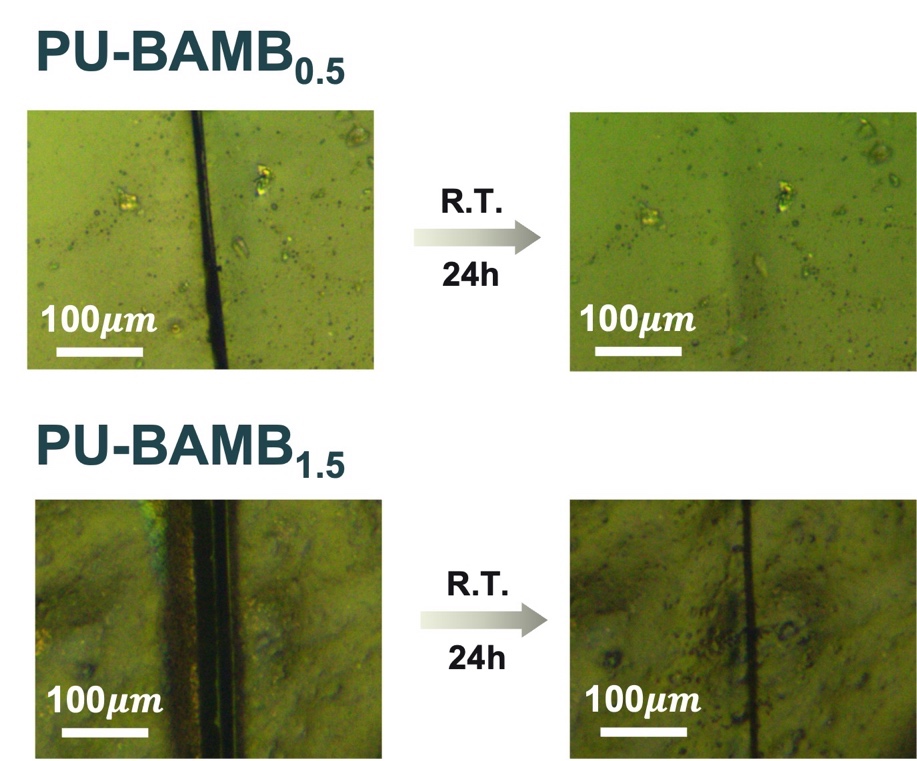
**

**Figure S4** Self-healing scratch optical images of PU-BAMB_0.5_ and PU-BAMB_1.5._


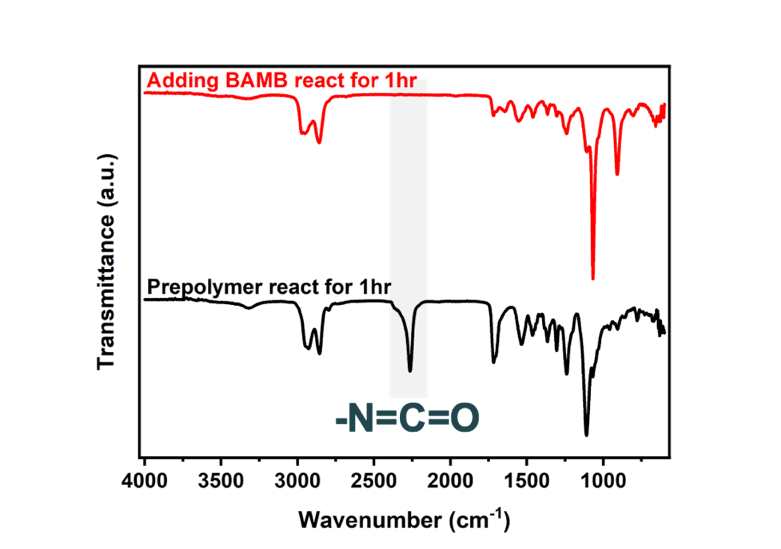


**Figure S5** FT-IR spectra for the polymerization process of PU-BAMB.

**Table S2** Molecular weight and polymer dispersity index (PDI) of prepolymer, PU-BAMB, PU-CH, PU-NOR, and PU-TPA.

| Sample Name | M_n_ | M_w_ | PDI |
| --- | --- | --- | --- |
| Prepolymer | 2,282 | 2,785 | 1.22 |
| PU-BAMB | 4,618 | 15,751 | 3.41 |
| PU-CH | 3,482 | 12,357 | 3.55 |
| PU-NOR | 4,310 | 15,554 | 3.61 |
| PU-TPA | 5,284 | 12,581 | 2.38 |


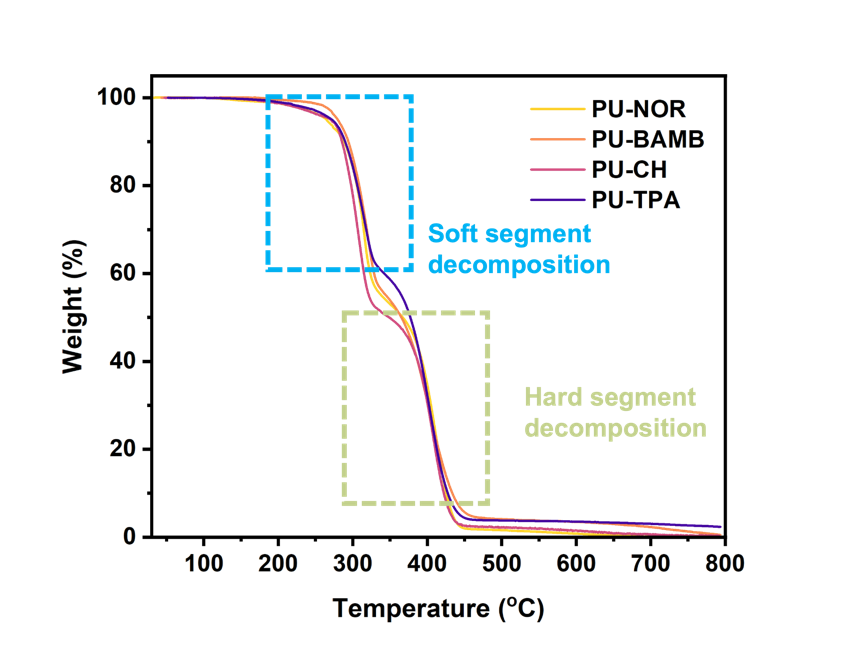


**Figure S6** Thermogravimetric analysis of PU-NOR, PU-BAMB, PU-CH, and PU-TPA.

**Table S3** Summary of thermal decomposition temperature (T_d_) of PU-BAMB, PU-CH, PU-NOR, and PU-TPA.

|  | PU-BAMB | PU-CH | PU-NOR | PU-TPA |
| --- | --- | --- | --- | --- |
| T_d_ (˚C) | 281.7 | 270.4 | 272.5 | 272.1 |


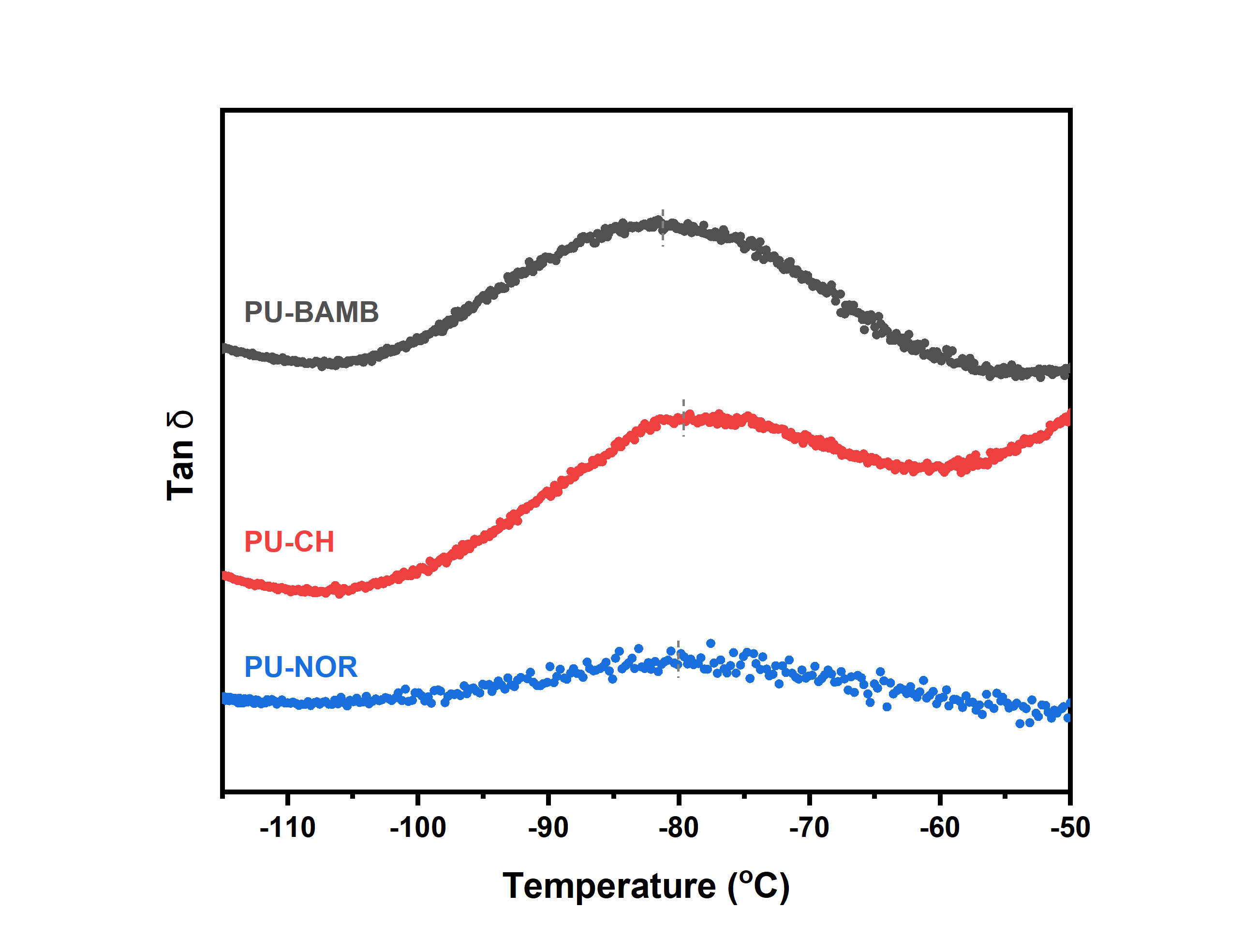


**Figure S7** Dynamic mechanical analyzer (DMA) spectra of PU-BAMB, PU-CH, and PU-NOR.

**Table S4** Summary of glass transition temperature (T_g_) of PU-NOR, PU-BAMB, and PU-CH.

|  | PU-BAMB | PU-CH | PU-NOR |
| --- | --- | --- | --- |
| T_g_ (˚C) | -81.26 | -79.5 | -80.09 |


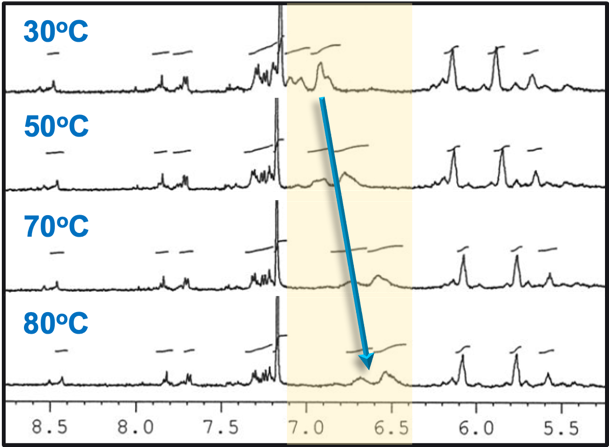


**Figure S8** Variable-temperature ^1^H NMR spectra of PU-BAMB.


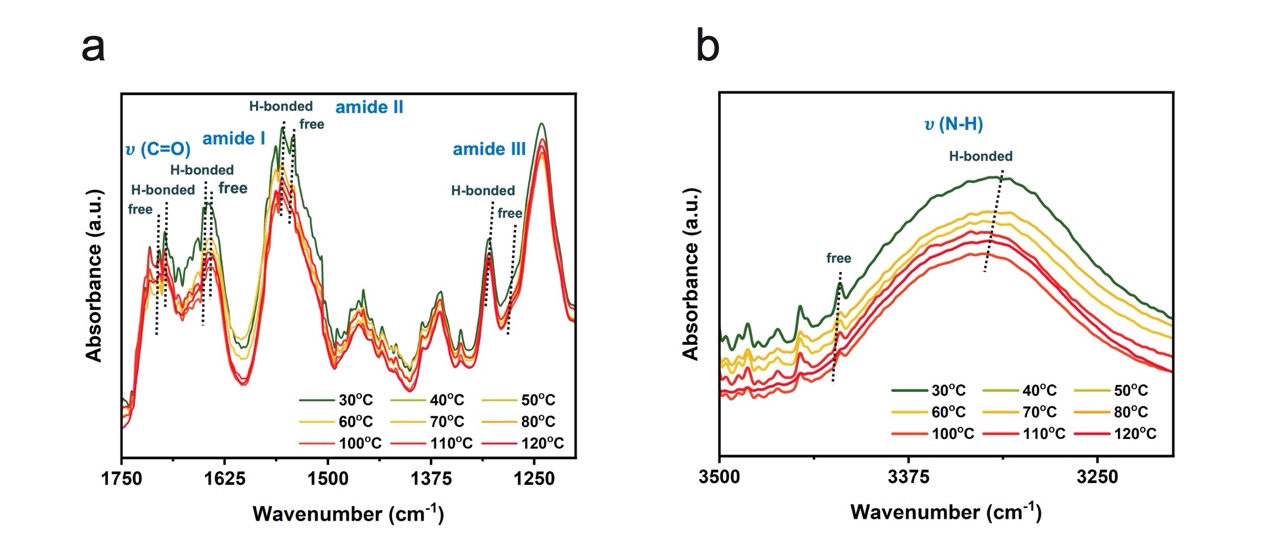


**Figure S9** Variable-temperature FT-IR spectra of PU-BAMB (a) 1250–1750 cm⁻¹ and (b) 3250–3500 cm⁻¹.

**Figure S10** Stress–strain curves of PU-TPA
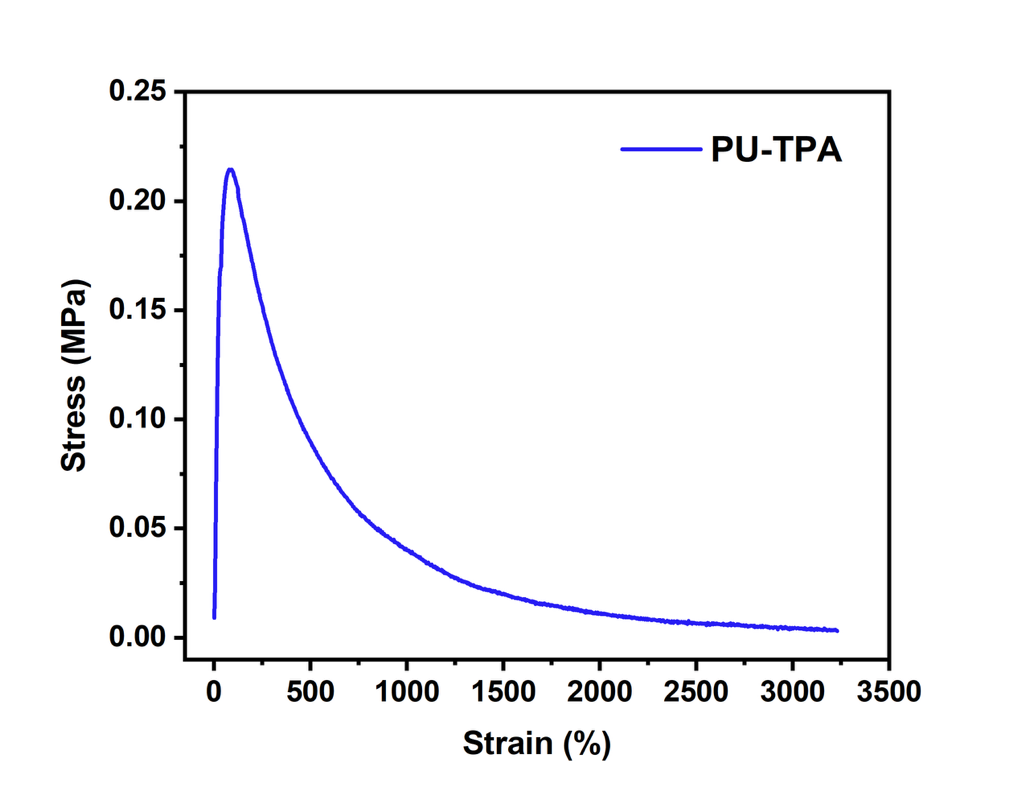
.

**Table S5** Summary of the mechanical properties of PU-BAMB, PU-CH, PU-NOR, and PU-TPA.

|  | Tensile Strength (MPa) | Elongation at break (%) | Modulus (MPa) | Toughness  (MJ m^-3^) |
| --- | --- | --- | --- | --- |
| PU-BAMB | 21.08 | 1056 | 9.46 | 149.64 |
| PU-CH | 7.31 | 790 | 2.54 | 41.55 |
| PU-NOR | 3.59 | 732 | 7.79 | 18.33 |
| PU-TPA | 0.21 | 3230 | 0.83 | 1.33 |


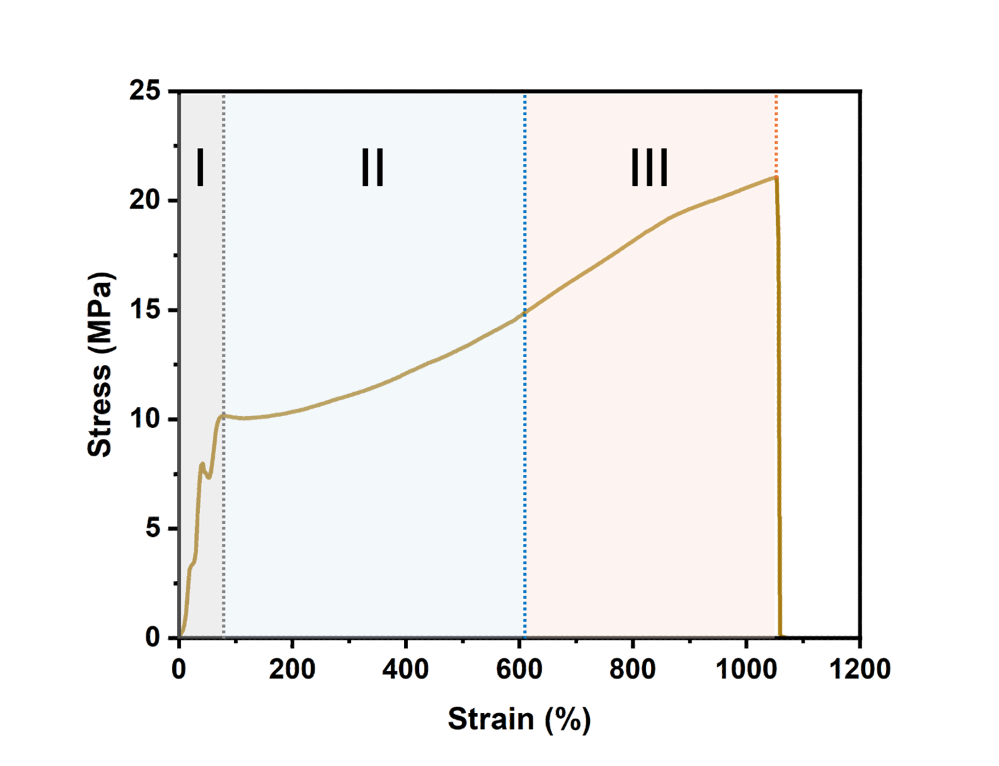


**Figure S11** Nonlinear stress–strain curve of PU-BAMB.


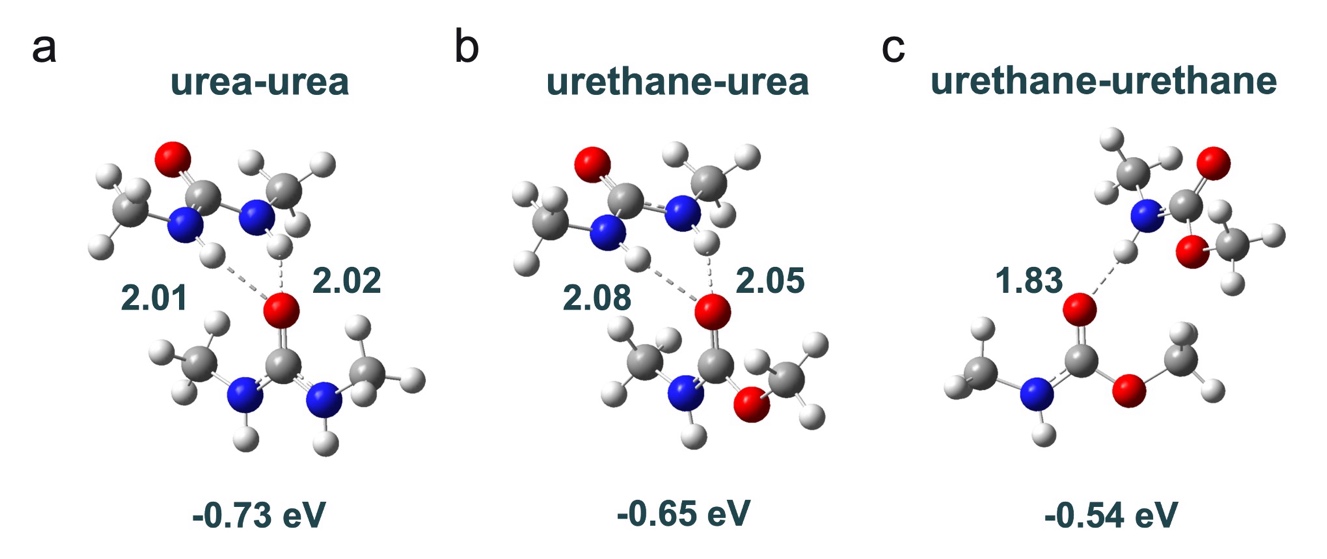


**Figure S12** Binding energies (E_bind_) of hard segments in the polymer system (a) urea–urea, (b) urethane–urea, and (c) urethane–urethane. E_bind_ is calculated using the equation: E_bind_ = E_complex_ – ∑E_molecule_, where E_complex_ is the total energy of the complex, and E_molecule_ is the total energy of the isolated molecules.


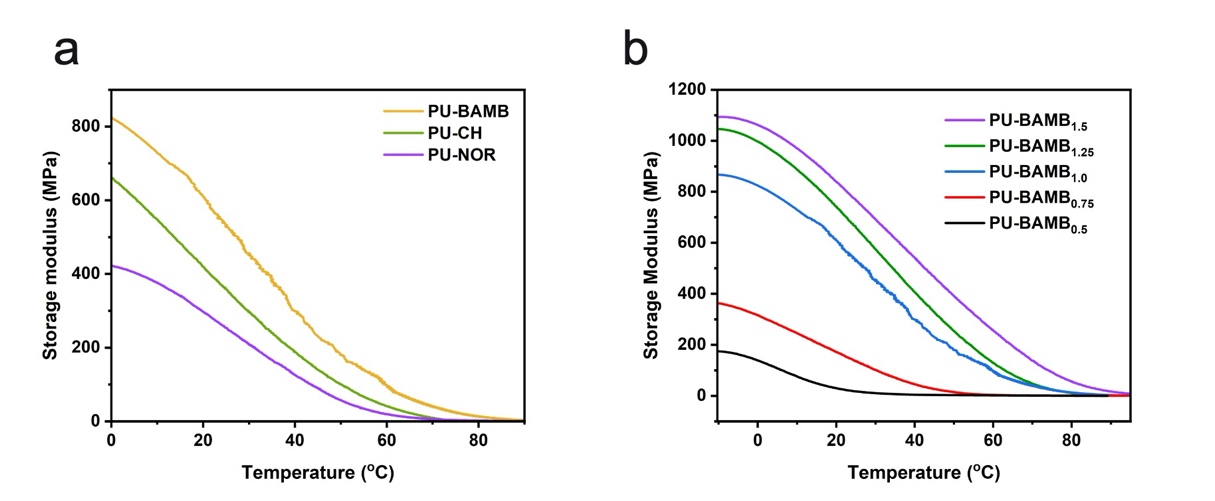


**Figure S13** (a) DMA storage modulus curve of the elastomers (b) DMA storage modulus curves of elastomers with different BAMB feed ratios.

**
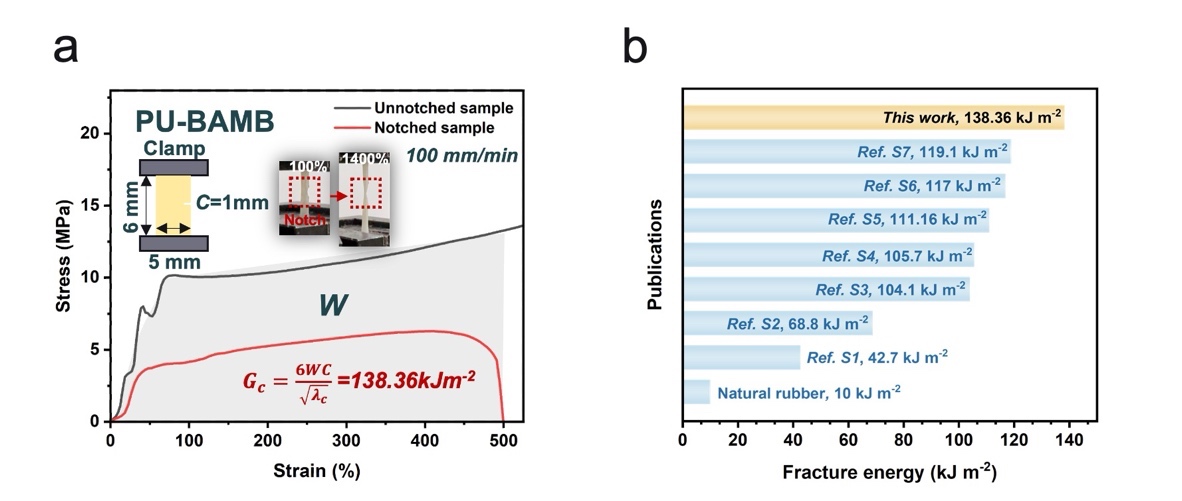
**

**Figure S14** (a) Stress–strain curves of notched and unnotched PU-BAMB elastomers used for fracture energy calculation, with inset photographs showing the stretching process. (b) Comparison of the fracture energy of PU-BAMB with previously reported materials^[4]^.

**Table S6** Summary and comparison of fracture energies with reported materials in the literature.

| Sample | Fracture energy  (kJ m^-2^) | Ref. |
| --- | --- | --- |
| Natural rubber | ~10 | - |
| PPGTD-IDA | 42.7 | [4] a |
| PU_2.1_–1.2 | 68.8 | [4] b |
| PDM-2.5 | 104.1 | [4] c |
| PU-S8L2 | 105.7 | [4] d |
| SSPUGIT- | 111.16 | [4] e |
| PUMI-A_3_D_7_/Al(OH)_3_/GP | 117 | [4] f |
| PU_3.8-80/20_ | 119.1 | [4] g |
| **PU-BAMB** | **138.36** | **This work** |


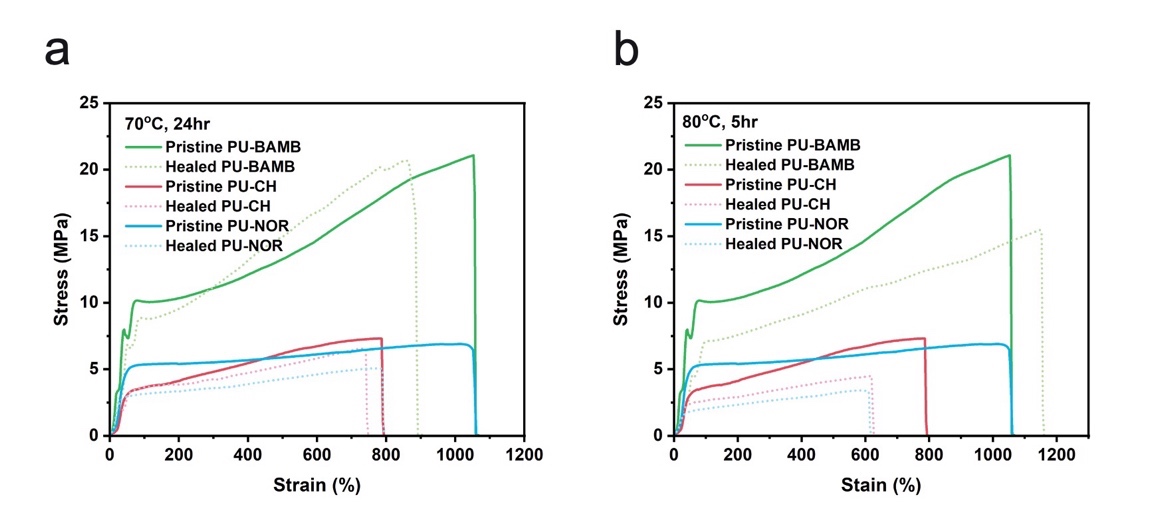


**Figure S15** (a) Stress–strain curves of all elastomers after healing at 70 °C for 24 h (b) Stress–strain curves of all elastomers after healing at 80 °C for 5 h.

**Table S7** Summary of self-healing efficiencies under different conditions.

|  | PU-BAMB | PU-CH | PU-NOR |
| --- | --- | --- | --- |
| 90˚C, 1hr | 96.5 ± 2.3 | 51.8 ± 13.7 | 16.3 ± 5.7 |
| 80˚C, 5hr | 80.3 ± 4.5 | 36.9 ± 11.2 | 13.3 ± 2.9 |
| 70˚C, 24hr | 81.2 ± 5.6 | 73.7 ± 6.2 | 45.0 ± 7.2 |


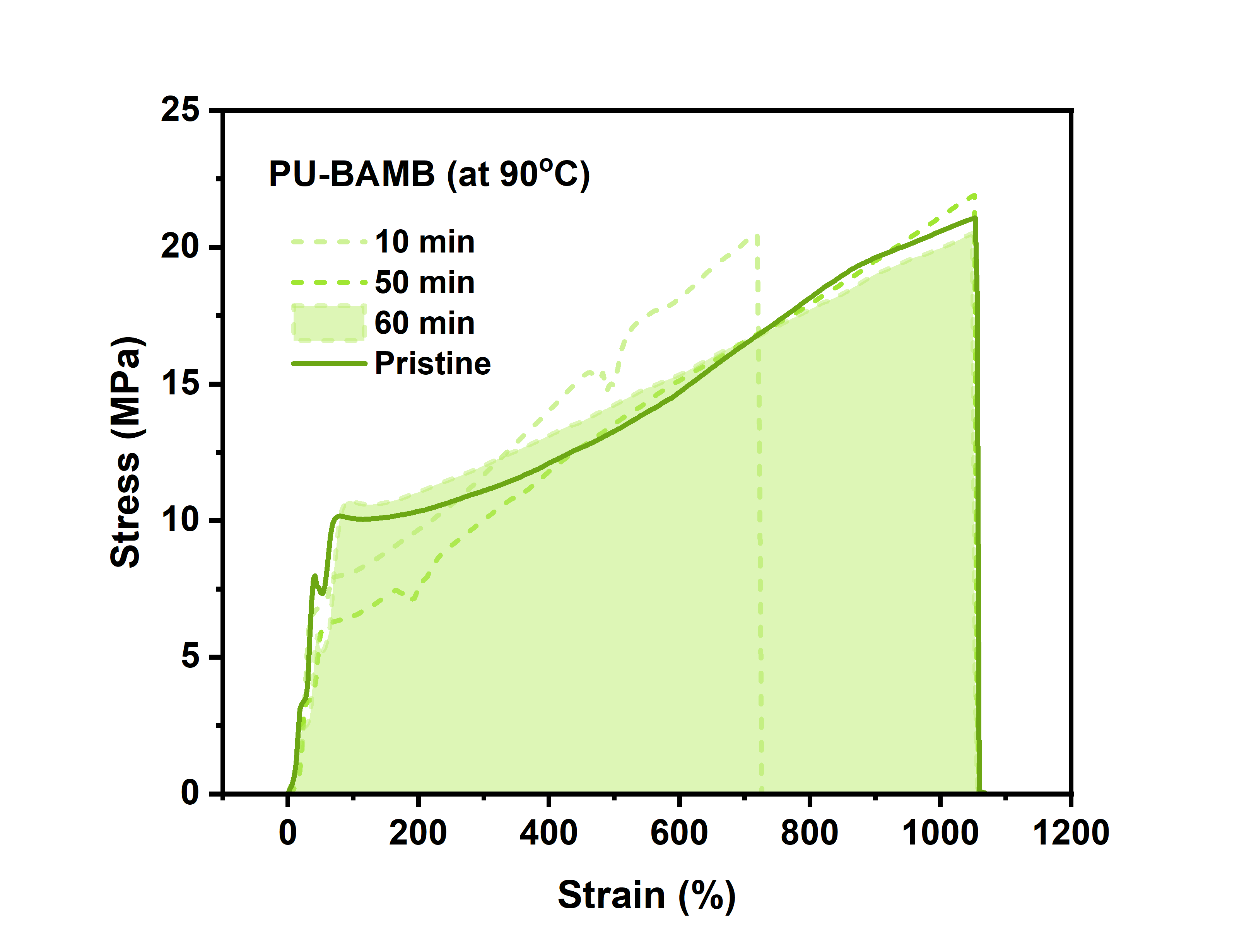


**Figure S16** Stress–strain curves of pristine and healed PU-BAMB elastomers at different healing times at 90 °C.

**Table S8** Summary of self-healing efficiency of PU-BAMB elastomer at 90 °C.

|  | Tensile strength (MPa) | Elongation at break (%) | Modulus (MPa) | Toughness (MJm^-3^) | Efficiency (%) by toughness |
| --- | --- | --- | --- | --- | --- |
| Pristine | 21.08 | 1056 | 14.72 | 149.64 | - |
| 90˚C, 10min | 20.55 | 722 | 5.95 | 93.94 | 62.8 |
| 90˚C, 50min | 21.90 | 1055 | 4.11 | 142.1 | 95.0 |
| 90˚C, 1hr | 20.51 | 1053 | 8.49 | 145.2 | 96.5 |


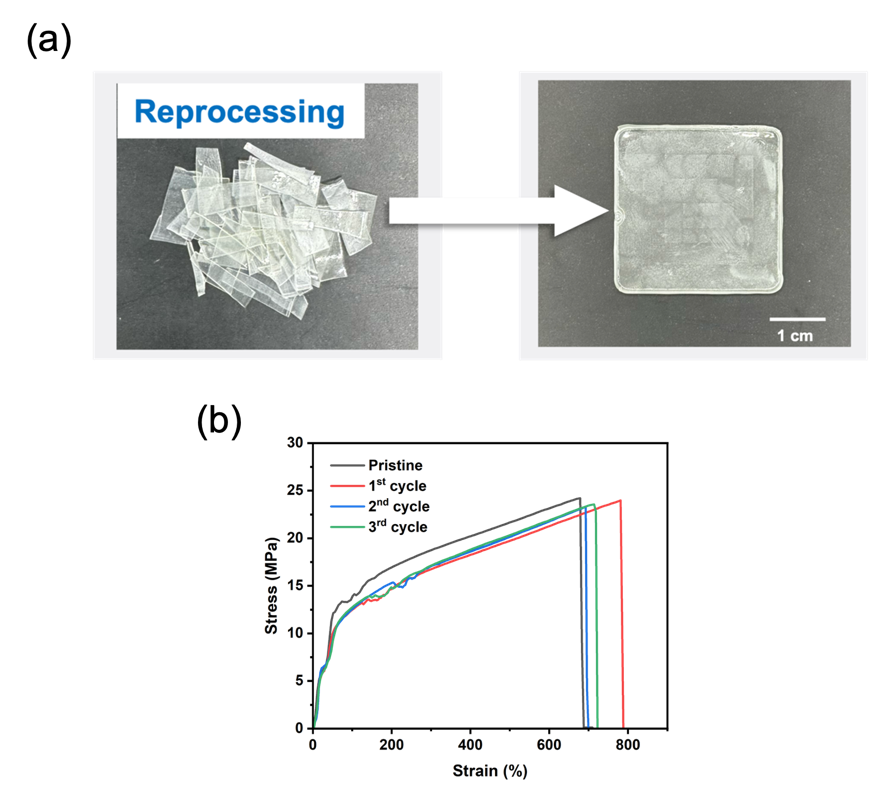
 **Figure S17 (a)** The solvent recycling process of the PU-BAMB elastomer. (b) Stress–strain curves of the PU-BAMB elastomer for the first, second and third cycles of the recycling test.


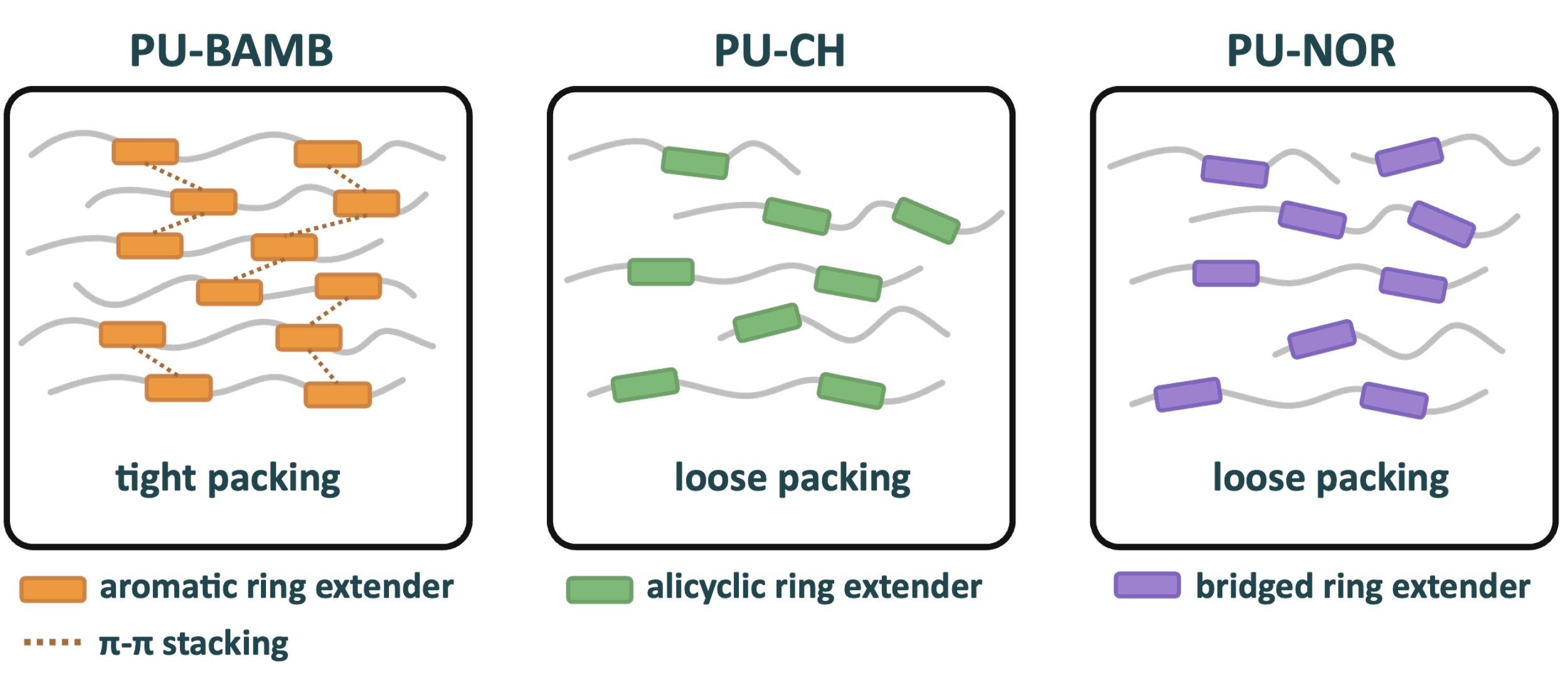
**Figure S18** Schematic illustration of the chain segment interaction mechanisms in PU-BAMB, PU-CH, and PU-NOR.


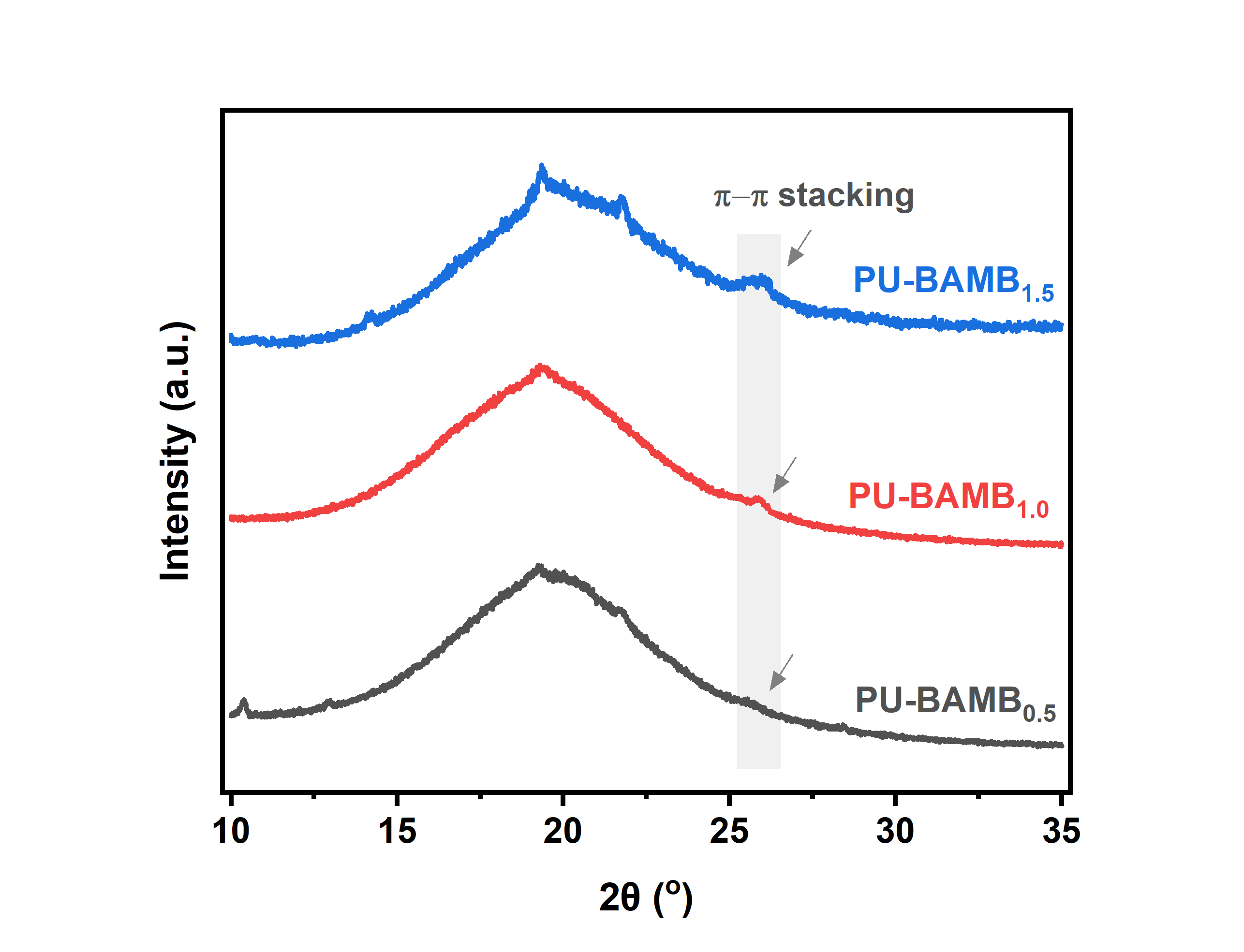


**Figure S19** XRD spectra of PU-BAMB_1.5_, PU-BAMB_1.0_, and PU-BAMB_0.5_.

**
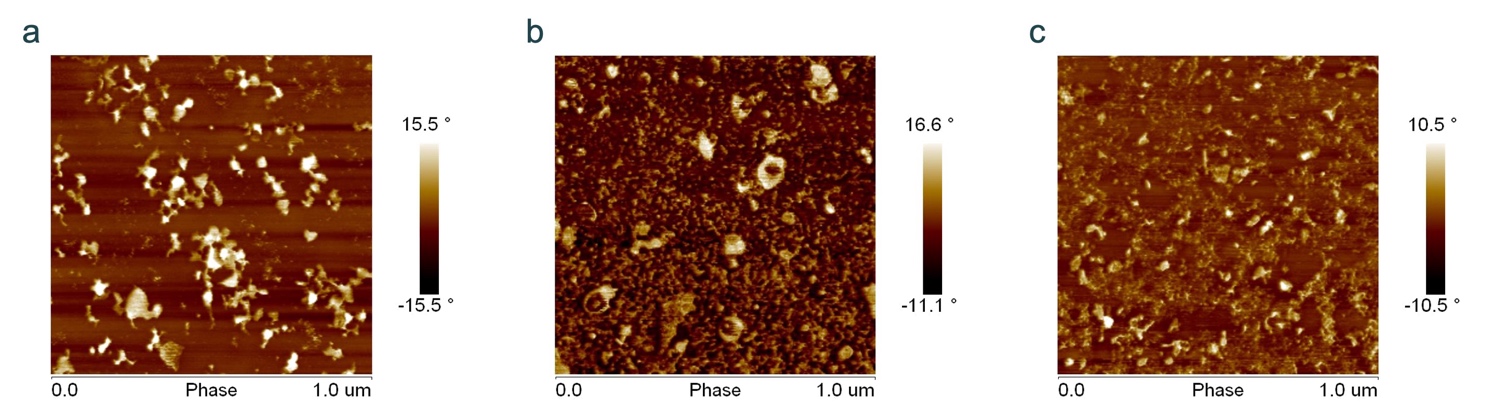
**

**Figure S20** AFM phase images (1 × 1 µm², tapping mode) of (a) PU-BAMB, (b) PU-CH, and (c) PU-NOR.


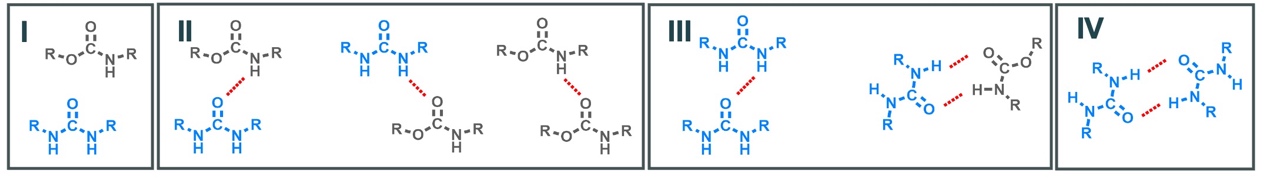


**Figure S21** Types of deconvoluted sub-peaks of the C=O absorption band in FT-IR spectra.

**Table S9** Summary of assignments of deconvoluted sub-peaks in the FT-IR C=O stretching vibration region of the elastomers.

| **Assignment** | | **Wavenumber (cm^-1^)** | | | **Area (%)** | | |
| --- | --- | --- | --- | --- | --- | --- | --- |
|  |  | **PU-BAMB** | **PU-CH** | **PU-NOR** | **PU-BAMB** | **PU-CH** | **PU-NOR** |
| $\upsilon$(C=O)  urethane | Free | I (1721) | I (1720) | I (1719) | 8.65 | 24.64 | 17.30 |
|  | H-bonded  (Ordered) | II (1700) | II (1699) | II (1697) | 34.10 | 17.01 | 30.07 |
| $\upsilon$(C=O)  urea | H-bonded  (Disordered) | III (1665) | III (1661) | III (1658) | 10.07 | 26.03 | 31.89 |
|  | H-bonded  (Ordered) | IV (1642) | IV (1635) | IV (1635) | 47.18 | 32.32 | 20.74 |
| **Total degree of bonded  H-bond** | - | | | | **91.35** | **75.36** | **82.70** |


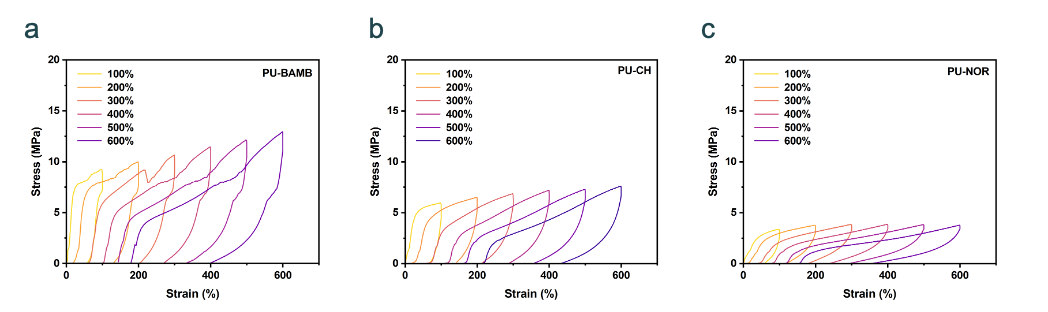


**Figure S22** Cyclic loading/unloading curves of (a) PU-BAMB, (b) PU-CH, and (c) PU-NOR with 100~600% tensile strains and there was no waiting time between two cyclic tensile tests.


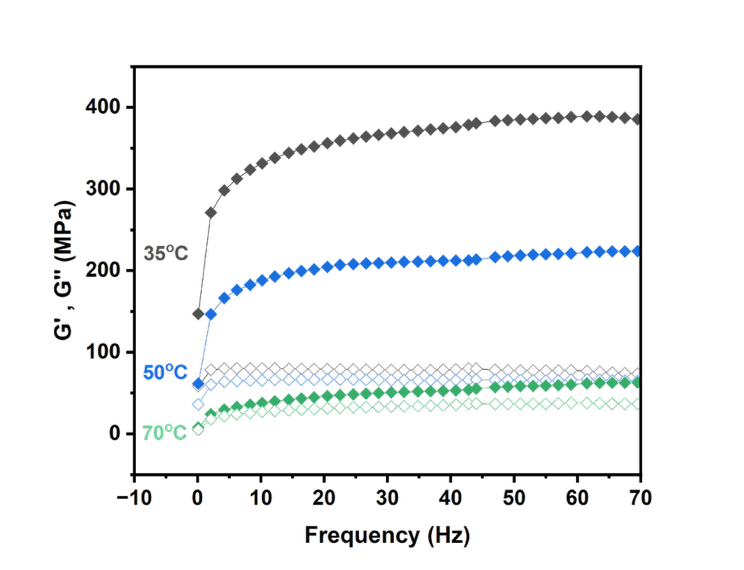
**Figure S23** Storage modulus (G’) and loss modulus (G”) of PU-BAMB in the frequency range of 0.01–70 Hz.

**Table S10** Summary and comparison of impact-resistance values with previously reported materials^[5]^.

| Material | Maximum stress  (MPa) | Strain rate  (s^-1^) | Ref. |
| --- | --- | --- | --- |
| **PU-BAMB** | **87.27 (at 90% strain)** | **0.09 s^-1^** | **This work** |
|  | **60.01 (at 90% strain)** | **0.06 s^-1^** |  |
|  | **28.7 (at 90% strain)** | **0.01 s^-1^** |  |
|  | **12.36 (at 90% strain)** | **0.002 s^-1^** |  |
| polyTA/arginine SPNs | 20 (at 90% strain) | 0.1 s^-1^ | *J. Am. Chem. Soc.* 2024, 146, 7533−7542 |
|  | 15.3 (at 90% strain) | 0.06 s^-1^ |  |
|  | 5 (at 90% strain) | 0.01 s^-1^ |  |
|  | 2.5 (at 90% strain) | 0.002 s^-1^ |  |
| SPM | 1.3 (at 80% strain) | 0.16 s^-1^ | *J. Am. Chem. Soc.* 2021, 143, 2, 1162–1170 |
|  | 0.21 (at 80% strain) | 0.016 s^-1^ |  |
|  | 0.21 (at 80% strain) | 0.0016 s^-1^ |  |
| PEE3 | 8.2 (at 60% strain) | ~0.002 s^-1^ | *Nat. Mater.* 2024, 23.8: 1107-1114. |
| PIL DN | 15 (at 60% strain) | ~0.2 s^-1^ | ***Adv. Mater.*** 2024, 36.13: 2311214. |
| IPU-30BC | 7 (at 50% strain) | 0.1 s^-1^ | ***Mater. Horiz.*,** 2024,**11**, 3143-3156 |
|  | 3.3 (at 50% strain) | 0.01 s^-1^ |  |
| LC-semi-IPN | 45 (at 80% strain) | 0.128 s^-1^ | *Nat. Commun.* 2024, 15.1: 9902. |
|  | 32 (at 80% strain) | 0.032 s^-1^ |  |
|  | 26 (at 80% strain) | 0.008 s^-1^ |  |
|  | 24 (at 80% strain) | 0.002 s^-1^ |  |
| P(HMA-*co*-ViCL) | 2.8 (at 60% strain) | 0.11 s^-1^ | *Angew. Chem. Int. Ed.* 2024, 63, e202406937*.* |
|  | 1.1 (at 60% strain) | 0.05 s^-1^ |  |
| PolyPOSS_29_ | 4 (at 50% strain) | 0.1 s^-1^ | *Angew. Chem. Int. Ed.* 2021, 60, 22212. |
|  | 1.2 (at 50% strain) | 0.03 s^-1^ |  |
|  | 1 (at 50% strain) | 0.016 s^-1^ |  |
|  | 0.4 (at 50% strain) | 0.003 s^-1^ |  |
| DCPFG | 0.045 (at 40% strain) | 0.125 s^-1^ | *Angew. Chem. Int. Ed.* 2024, *63*, e202401845. |
|  | 0.03 (at 40% strain) | 0.025 s^-1^ |  |
|  | 0.015 (at 40% strain) | 0.005 s^-1^ |  |
|  | 0.01 (at 40% strain) | 0.001 s^-1^ |  |

**
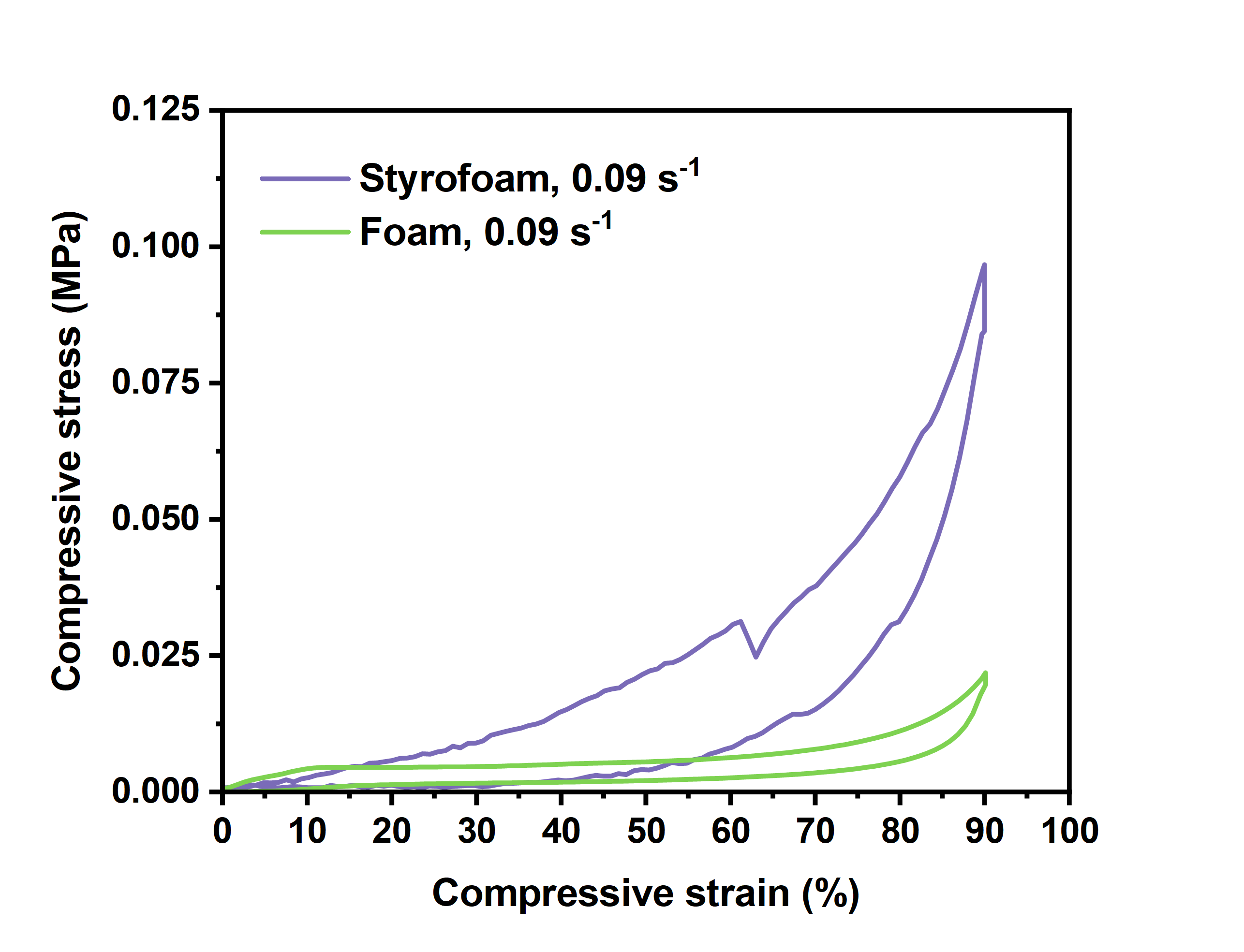
**

**Figure S24** Cyclic compression performance of Styrofoam and sponge foam at a strain rate of 0.09 s⁻¹.


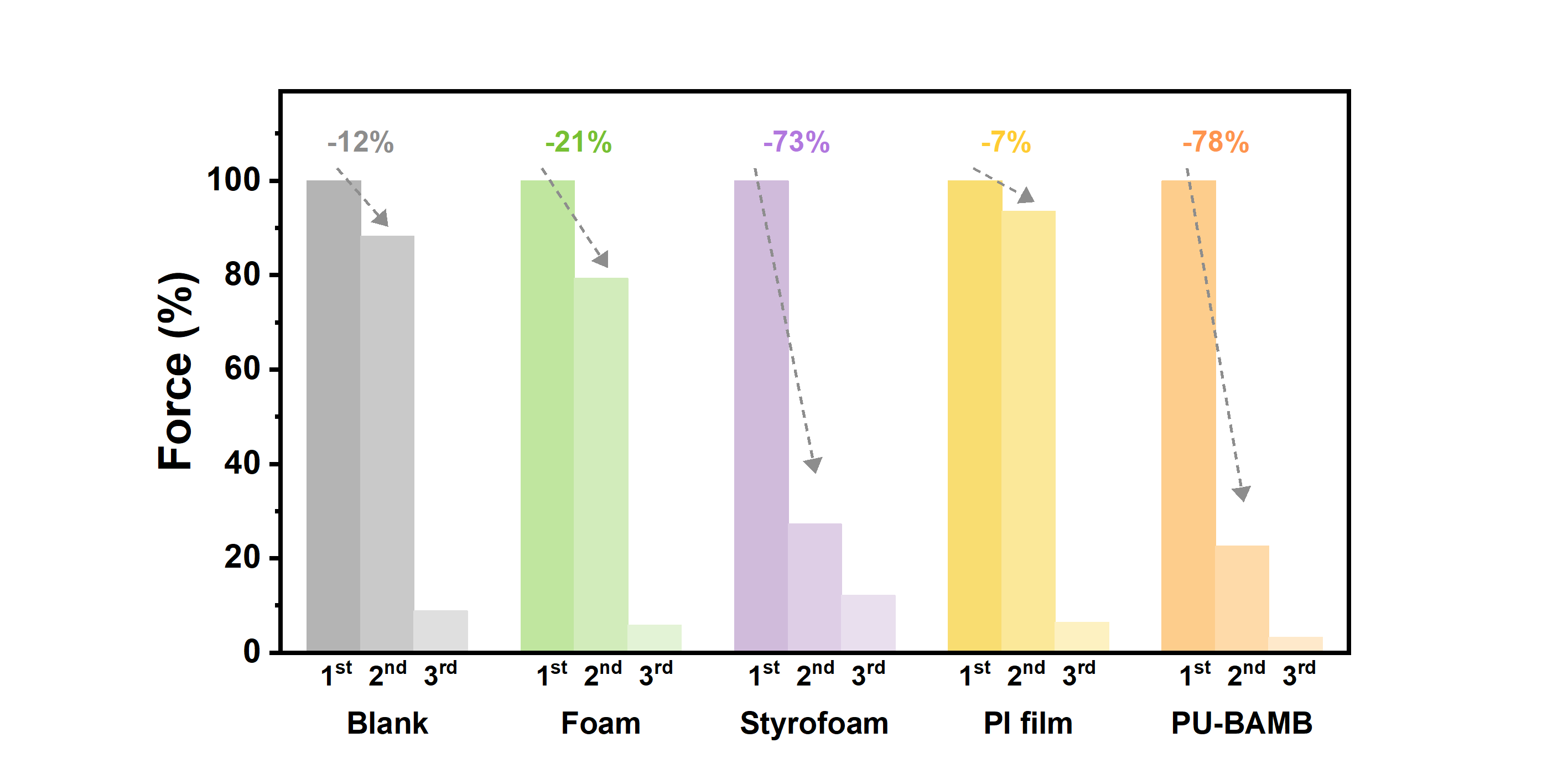
**Figure S25** Impact force attenuation ratios of PU-BAMB and commercial materials.

**Reference**

[1] M. Frisch, G. Trucks, H. Schlegel, G. Scuseria, M. Robb, J. Cheeseman, G. Scalmani, V. Barone, G. Petersson, H. Nakatsuji, *Wallingford, CT* **2016**.

[2] a)A. D. Becke, *Physical review A* **1988**, 38, 3098; b)C. Lee, W. Yang, R. G. Parr, *Physical review B* **1988**, 37, 785.

[3] S. Grimme, S. Ehrlich, L. Goerigk, *J. Comput. Chem.* **2011**, 32, 1456.

[4] a)D. Wang, J. Xu, J. Chen, P. Hu, Y. Wang, W. Jiang, J. Fu, *Adv. Funct. Mater.* **2020**, 30, 1907109; b)Y. Luo, J. Chen, G. Situ, C. Li, C. Zhang, F. Li, C.-H. Li, Z. Luo, X. Zhang, *Chem. Eng. J.* **2023**, 469, 143958; c)Y. Li, W. Li, A. Sun, M. Jing, X. Liu, L. Wei, K. Wu, Q. Fu, *Mater. Horiz.* **2021**, 8, 267; d)T. Jing, X. Heng, T. Jingqing, L. Haozhe, L. Li, L. Pingyun, G. Xiaode, *Chem. Eng. J.* **2023**, 465, 142887; e)F. Sun, L. Liu, T. Liu, X. Wang, Q. Qi, Z. Hang, K. Chen, J. Xu, J. Fu, *Nat. Commun.* **2023**, 14, 130; f)H. Xu, J. Ji, H. Li, J. Tu, Z. Fan, X. Zhang, X. Guo, *Chem. Eng. J.* **2023**, 475, 146018; g)L. Xia, H. Tu, W. Zeng, X. Yang, M. Zhou, L. Li, X. Guo, *J. Mater. Chem. A* **2022**, 10, 4344.

[5] a)K. Liu, L. Cheng, N. Zhang, H. Pan, X. Fan, G. Li, Z. Zhang, D. Zhao, J. Zhao, X. Yang, *J. Am. Chem. Soc.* **2020**, 143, 1162; b)J. F. Yin, H. Xiao, P. Xu, J. Yang, Z. Fan, Y. Ke, X. Ouyang, G. X. Liu, T. L. Sun, L. Tang, *Angew. Chem.* **2021**, 133, 22386; c)J. Cheng, X. Yao, Z. Zhang, Y. Tan, N. Hu, C. Ma, G. Zhang, *Mater. Horiz.* **2024**, 11, 3143; d)L. Cheng, J. Zhao, Z. Xiong, S. Liu, X. Yan, W. Yu, *Angew. Chem.* **2024**, 136, e202406937; e)Y. He, Y. Cheng, C. Yang, C. F. Guo, *Nat. Mater.* **2024**, 23, 1107; f)Q. Li, W. Li, Z. Liu, S. Zheng, X. Wang, J. Xiong, F. Yan, *Adv. Mater.* **2024**, 36, 2311214; g)H. Qiao, B. Wu, S. Sun, P. Wu, *J. Am. Chem. Soc.* **2024**, 146, 7533; h)Z. Yang, Y. Yang, H. Liang, E. He, H. Xu, Y. Liu, Y. Wang, Y. Wei, Y. Ji, *Nat. Commun.* **2024**, 15, 9902.
